# Kinetic Investigation of Tobermorite Synthesis for the Recovery of Carcinogenic Respirable Crystalline Silica (RCS)

**DOI:** 10.1021/acsomega.5c06547

**Published:** 2025-10-24

**Authors:** Daniele Malferrari, Giulio Galamini, Maddalena Bernini, Riccardo Fantini, Giulia Malvolti, Alessandro F. Gualtieri

**Affiliations:** † Department of Chemical and Geological Sciences, 9306 University of Modena and Reggio Emilia, Via G. Campi 103, Modena I-41125, Italy; ‡ Inter-Departmental Research and Innovation Centre on Construction and Environmental Services of the University of Modena and Reggio Emilia, Via P. Vivarelli 10, Modena I-41125, Italy

## Abstract

Respirable crystalline silica (RCS), a hazardous byproduct of quartzite processing, poses severe occupational and environmental health risks. To address both waste valorization and health concerns, this study developed an end-of-waste strategy for converting quartz-rich quarry dust (QD) into substituted tobermorite under mild hydrothermal conditions. A systematic series of syntheses was carried out at 120, 130, and 140 °C under dynamic conditions using controlled mixtures composed of QD together with KRY·AS (a material derived from the thermal inertization of cement asbestos) or CaO as calcium sources and a small amount (2.5 wt %) of phillipsite-rich zeolitic tuff as a catalytic additive. Crystallization pathways and reaction kinetics were analyzed through X-ray diffraction, scanning electron microscopy, and thermogravimetric/thermodifferential methods. Results showed that quartz dissolution is the primary source of silica for tobermorite crystallization, which proceeds according to first-order kinetics. The apparent activation energies derived from Arrhenius plots were 101 ± 33 and 111 ± 34 kJ mol^–1^ depending on the Ca source (KRY·AS or CaO, respectively). When using KRY·AS, the coexistence of katoite and amorphous calcium silicate hydrate phases indicated competing reaction pathways, while CaO favored more direct quartz-to-tobermorite conversion. Optimal tobermorite yields were achieved at 140 °C, reaching nearly 48 wt % in CaO-based mixtures. Lower temperatures led to slower growth and persistence of amorphous calcium silicate hydrate phases, whereas elevated temperatures favored rapid and more complete conversion with reduced secondary phase formation. Thermal analyses corroborated these findings, evidencing stable tobermorite and extensive carbonate formation. Scanning electron microscopy further confirmed the complete consumption of quartz, including respirable fractions, validating the process as compliant with end-of-waste criteria. Beyond silica detoxification, carbonate phases consistently formed, suggesting the potential dual benefit of hazardous waste valorization and incidental CO_2_ sequestration. Taken together, these results highlight a novel, low-energy valorization route for RCS-containing waste, advancing circular economy goals while offering prospects for both functional material production and carbon capture applications.

## Graphical abstract

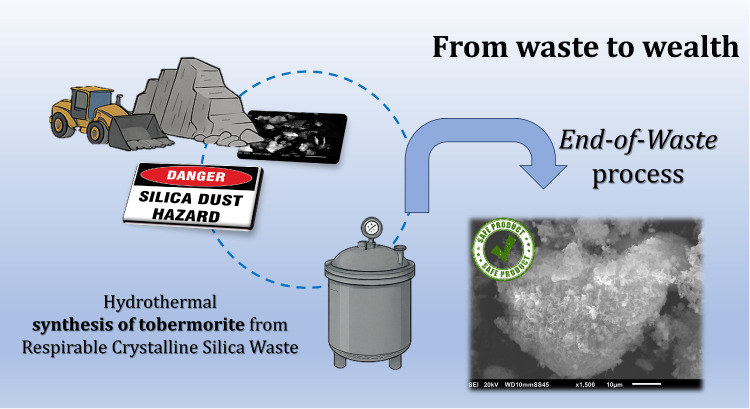

## 1 Introduction

Crystalline silica (SiO_2_), most commonly occurring as quartz, is a widespread mineral present in various rocks, as well as in byproducts of materials processing and/or disposal. Humans are routinely exposed to silica nanoparticles from a variety of backgrounds, both environmental and occupational. While silica is not chemically hazardous, chronic inhalation of fine crystalline silica particles can pose significant health risks, including increased incidence risk of chronic obstructive pulmonary disease, tuberculosis, cancer, and pulmonary fibrosis, clinically referred to as silicosis.^1–4^ The primary hazard is associated with the generation of respirable crystalline silica (RCS), defined as crystalline silica particles with an aerodynamic diameter smaller than 10 μm.^5^ These fine dust particles are typically produced during activities involving the mechanical breakdown of crystalline silica-containing materials (e.g., concrete, stone, sand) through processes such as cutting, grinding, drilling, or crushing. The risk is significant when airborne RCS concentrations exceed the threshold of 0.1 mg/m.^3,5–10^

To circumscribe the damages from RCS, regulatory agencies and occupational health bodies instituted measures such as personal protective equipment requirements and exposure limits.^10–12^ Nevertheless, there is an additional issue that must necessarily be addressed: the disposal of large quantities of waste containing RCS, as landfill storage is an option that is no longer sustainable. Therefore, one of the main challenges facing scientific research is the development of technologies and production processes, named end-of-waste,^13,14^ that involve the use, and thus recovery, of waste or byproducts, thereby transforming them into resources.

While various silica-containing waste streams, including spent solar panels,^15^ fly and bottom ash,^16–18^ construction scrap,^19^ and lake and river sediments,^20–26^ have been explored for reuse, their potential RCS content and, crucially, its postreuse residue were often overlooked. Moreover, byproducts from natural stone processing (e.g., grinding, cutting, or drilling residues), which likely contain the highest RCS concentrations, have largely been disregarded. This oversight is probably due to the heterogeneous distribution of these materials (often processed at quarry sites) and a significant lack of knowledge regarding their mineralogical composition and associated hazards.^9,27–31^ Some of these materials, as in the case presented, are largely composed of quartz with particle sizes under 10 μm, warranting their classification as hazardous waste. Despite this, they show potential for synthesizing nanoporous materials, with tobermorite ranking as a strong candidate due to its favorable properties, such as medium to high surface area,^32^ high adsorption and cation exchange capacity,^31,33–41^ and low synthesis energy requirements.^38,42,43^

Tobermorite is a member of a group of calcium silicate hydrate minerals that closely resemble 2:1 swelling layer silicates. Within the tobermorite group, each member belongs to a family of polytypic compounds, which can be described using order/disorder theory.^44,45^ Although a new nomenclature has recently been introduced that proposes a tobermorite supergroup,^46,47^ most of the existing literature mainly focuses on three main polytypes, namely plombierite, tobermorite, and riversideite. These polytypes, also known as 14 Å-, 11 Å-, and 9 Å-tobermorite, respectively, are defined by their interlayer spacing (about 14.0, 11.3, and 9.30 Å), which is mainly influenced by the water content.^48^ Simplifying, without considering polytypism and disorder, the structure of tobermorite consists of a series of Ca–Si layers stacked along the *c*-axis. The layers consist of a central sheet of CaO-like polyhedra paired with wollastonite-like tetrahedral chains running along the *b*-axis. Water, solvating, and exchangeable cations—mostly Ca^2+^ in natural samples—are intercalated between the Ca–Si units in an amount depending on the Al^3+^ and/or Fe^3+^ for Si^4+^ substitutions in the so-called Al-substituted tobermorites (see below).

Tobermorite is rare in nature, but, as mentioned above, it can be easily synthesized under mild hydrothermal conditions. In particular, Al-substituted tobermorites, an 11 Å polytype also known as substituted tobermorite, can be synthesized from materials containing silicon, calcium, iron, and aluminum.^37^ Iron and aluminum act as possible isomorphic and heterovalent substituents for silicon,^37,38,49–51^ resulting in a charge deficit that enhances the mineral’s reactivity (adsorption and cation exchange capacity). These materials mostly include fly and bottom ash,^35,41,52–57^ paper recycling residues,^33,34,58,59^ glass,^43,60,61^ ash, and residue from organic material combustion.^42,62,63^ Nevertheless, other paths were explored using cement bypass dust,^64^ blast furnace slags,^40,65^ and mixtures of various materials.^38,59,66^ Mixing different materials turns out to be an advantageous strategy because it allows for optimizing the chemical conditions required for the synthesis of substituted tobermorite (Section 2.2) while simultaneously recovering different types of waste or scrap. As will be discussed in Section 2.2, several studies investigated how synthesis conditions influence tobermorite formation. These conditions include the Ca/Si molar ratio, aluminum content, agitation, water-to-solid ratio, temperature, time, and particle size of the source materials.

With these premises, an end-of-waste process was developed to recover fine rock dust (QD) resulting from quartzite processing and containing quartz in the form of RCS, to synthesize a substituted tobermorite-rich material. The process involves using not only QD but also KRY·AS,^67–69^ an end-of-waste material that has already proven useful in the synthesis of substituted tobermorite as Ca source.^38^ Yield maximization was investigated by analyzing crystallization kinetics under various experimental conditions. Crucially, each synthesis test was performed in parallel, substituting KRY·AS with CaO as the Ca source; this comparative approach made it possible to determine the effect of deviation from optimal synthesis conditions on process yield.

Unlike previous studies, this research targets crystalline quartz as the silica source, thus probing the kinetics and mechanisms of its dissolution and conversion under low-temperature hydrothermal conditions. A key question addressed in this work is therefore whether RCS-containing material can not only be reused but also detoxified through complete mineralogical transformation. Furthermore, by determining activation energies and reaction orders, this work provides mechanistic insights into the rate-limiting steps that control the transformation of crystalline silica into safe tobermorite, evaluating also the role of competing phases, such as amorphous calcium silicate hydrates, katoite, and carbonates, in influencing tobermorite yield and stability.

## 2 Analytical Methods and Materials

### 2.1 Materials

QD comes from the processing site of quarries located in northwest Italy. It is mainly generated from rock block cutting operations, and the amount produced is several thousand tons per year. KRY·AS derives from the thermal inertization of cement asbestos and is a material with cytotoxic effects comparable to commercial clinker.^67,68,70^ KRY·AS is therefore a safe material derived from an end-of-waste process that already finds use in some applications, such as porcelain stoneware slabs and foam glass;^70–72^ furthermore, it also has a chemical and mineralogical composition with limited variability.^69^ KRY·AS is particularly suitable for tobermorite synthesis because of its high CaO content formed during the treatment of cement asbestos. Besides QD and KRY·AS, a zeolitized tuff rich in phillipsite (ZT) was included, as phillipsite is known to catalyze tobermorite synthesis.^38,73^ ZT comes from a quarry near Grosseto (central Italy) and has a limited cost because it can be sourced as a byproduct. In fact, in the past, and to a large extent today, zeolitized tuffs were used to obtain natural stone for construction; over the years, significant amounts of zeolite-rich scrap have accumulated that can be profitably used in such applications.

### 2.2 Synthesis Procedure

Al-substituted tobermorite (hereafter referred to as tobermorite) can be easily synthesized in a simulated hydrothermal environment, provided that the molar ratios 0.80 < Ca/[Si + Al + Fe] < 0.85 and 0.00 < [Al + Fe]/[Al + Si + Fe] < 0.17 are approximately met.^34,49,51,64,74^ Tobermorite can also form with a different Ca/Si ratio; however, as the Ca/Si ratio increases, along with a greater presence of Al, the crystal morphology of tobermorite changes, an aspect that must necessarily be taken into account in applications.^75,76^ Based on the results described in the literature and mentioned above, a mixture (M1) was prepared by dosing 75, 22.5, and 2.5 wt % of KRY·AS (used as the main source of calcium), QD, and ZT, respectively (Table 1). A second mixture (M2) was prepared using pure CaO from Sigma-Aldrich as a source of Ca, replacing KRY·AS, by dosing 42.0, 55.5, and 2.5 wt % of CaO, QD, and ZT, respectively (Table 1). These mixtures were selected to meet the molar ratio described earlier and to maximize the recovery of QD. These materials, after equilibrating at room temperature for 24 h, were mixed in the amounts indicated using an agate mortar. The syntheses were carried out in Teflon-lined Parr autoclaves by adding 15 mL of 1 M NaOH (pure-grade reagent) solution to 1 g of each mixture; this ratio was found to perform best for fine particle starting materials.^38,77^ Although small particle sizes, particularly of quartz, can favor reaction kinetics,^77^ the starting material did not undergo further grinding processes as it was already small in size (see Section 3.1). The autoclaves were placed in an incubator on a tilting table (60 oscillations/min) to facilitate interaction between the solid and the solution, as agitation was found to improve tobermorite formation.^32,78^ The temperatures and interaction times were 120, 130, and 140 °C and 4, 8, 12, 24, 36, 48, 72, 108, and 144 h. Although the formation of tobermorite and other phases depends on a combination of multiple factors (e.g., reaction time, composition of the starting mixture, interaction mode, etc.), the temperature values of 120, 130, and 140 °C were chosen because they represent the range in which the formation of tobermorite is most favored.^79–87^ Below 120 and above 140 °C, tobermorite can form; nevertheless, below 120 °C (as well as at the beginning of the reaction), amorphous CSH (calcium silicate hydrate) phases are favored, whereas increasing temperature (or later in the reaction) leads to the formation of crystalline tobermorite or xonotlite, with xonotlite being favored above 140 °C.^50,87^ Moreover, above 150 °C, Al^3+^ for Si^4+^ substitutions, which drive the cation exchange capacity of the material, are more limited.^87^ Also, with a view to possible industrial scale-up, lower temperatures mean greater energy savings. Once the autoclaves were removed from the incubator and cooled, the solid was separated from the solution by centrifuging for 5 min at 6000 rpm and then washed with distilled water until a pH close to neutrality was obtained and air-dried. Each sample was then named by indicating the starting mixture (M1 or M2), temperature, and synthesis time in the label. For example, sample M1-72H-120 indicates a synthesis obtained at 120 °C starting from the M1 mixture after 72 h of interaction.

**Table 1:** Chemical (mol/100g) and Mineralogical (Weight %) Composition of QD, KRY·AS, and ZT (Experimental) and of M1 and M2 (Calculated; See above)^a^

	QD	KRY·AS	ZT	M1	M2		QD	KRY·AS	ZT	M1	M2
**Si**	1.4075	0.4139	0.8778	0.6490	0.8031	Analcime	-	-	3.4(2)	0.1	0.1
**Al**	0.1748	0.0798	0.3238	0.1073	0.1051	Augite	-	-	3.9(4)	0.1	0.1
**Fe**	0.0088	0.0341	0.0482	0.0288	0.0061	Brownmillerite	-	6.4(1)	-	4.8	-
**Ti**	0.0015	0.0026	0.0054	0.0024	0.0010	Calcite	trace	5.8(1)	-	4.4	-
**P**	0.0020	0.0000	0.0008	0.0005	0.0011	Chabazite	-	-	21.1(4)	0.5	0.5
**Mn**	0.0002	0.0010	0.0021	0.0008	0.0001	Larnite	-	44.6(5)	-	33.5	-
**Mg**	0.0107	0.2363	0.0270	0.1803	0.0066	Microcline	3.3(1)	-	-	0.7	1.8
**Ca**	0.0020	0.8622	0.0794	0.6491	0.7519	Muscovite	18.2(3)	-	3.3(3)	4.2	10.2
**Na**	0.0023	0.0083	0.0323	0.0075	0.0021	Periclase	-	6.4(1)	-	4.8	-
**K**	0.0625	0.0062	0.1232	0.0218	0.0377	Phillipsite	-	-	32.4(6)	0.8	0.8
**LOI**	1.99	9.65	13.94	7.88	1.45	Plagioclase	-	-	9.7(5)	0.2	0.2
						Quartz	78.3(9)	-	-	17.6	43.5
**Ca/[Si + Al + Fe]**				0.8268	0.8224	Sanidine	-	-	13.1(6)	0.3	0.3
**[Al + Fe]/[Al + Si + Fe]**				0.1732	0.1216	Vaterite	-	2.4(2)	-	1.8	-
						Amorphous	0.2(1)	34.5(6)	13.1(9)	26.2	0.4
						CaO (addition)	-	-	-	-	42.0
						χ^2^	12.77	1.827	6.016	-	-
						*R*_p_	0.0889	0.0787	0.0605	-	-
						*R*_wp_	0.1195	0.0602	0.0716	-	-

a Chemical composition of KRY·AS and ZT are as reported in a previous study (reproduced from ao-2021-04193p, Copyright 2021, American Chemical Society).^38^ LOI is the loss on ignition (weight loss % at 1100 °C). The literature references for the structural models used in QPA are analcime,^90^ augite,^91^ brownmillerite,^92^ calcite,^93^ chabazite,^94^ larnite,^95^ microcline,^96^ muscovite,^97^ periclase,^98^ phillipsite,^99^ plagioclase,^100^ quartz,^101^ sanidine,^102^ and vaterite.^103^ See Table S1 and Supporting Information for the method used for error calculation (values in parentheses in QPA).

### 2.3 Analytical Methods

A consolidated protocol of analysis, based on X-ray diffraction, scanning electron microscopy, chemical analysis, particle size analysis, and thermogravimetric analysis, was applied. Details about the methods, instruments, and experimental conditions are provided in Supporting Information.

### 2.4 Crystallization Kinetics of Tobermorite

To investigate the reaction kinetics, the weight fractions of tobermorite vs time (h) were normalized by their respective maximum values and taken as the end of the reaction. This allowed for their conversion into α-time plots. Outliers were manually removed. Each α-time plot was fitted using the nonlinear regression approach,^88^ assuming that the reaction is single-step, and the data were fitted with a first-order kinetic equation:

α=1−exp[−kt]

where *k* = rate constant (in h^–1^ here); *t* = time (h). The rate constants *k* calculated from the analysis of the isothermal α-time plots were successively used in the Arrhenius equation:^89^

$$k=A\;\exp[-\frac{E_{a}}{\mathrm{RT}}]$$

where A = frequency factor; *E*_a_ = apparent activation energy; *R* = gas constant = 0.00831446261815324 kJK^–1^ mol^–1^; *T* = temperature in K. By plotting ln­(*k*) versus 1/*T*, *E*_a_ can be calculated from the slope of the first-order curve. The data analyses were performed using SigmaPlot for Windows version 12.0 (Systat Software, Inc.).

## 3 Results and Discussion

### 3.1 Material Characterization and Mixture Composition

Chemical and quantitative mineralogical phase analyses (QPA) of the pristine materials, as well as the compositions of M1 and M2, are shown in Table 1.

QPA evidence indicates a high quartz content for QD; this feature, accompanied by the results of particle size analyses (Figure 1) suggested that some of the quartz might be present in respirable form, a finding later confirmed by scanning electron microscopy with energy-dispersive X-ray spectroscopy (SEM-EDX) measurements (Figure S1). Moreover, the grain size analysis (Figure 1) shows that QD is predominantly composed of silt-sized particles, with 96.24% of the material falling within the 2–50 μm range (USDA classification) and 90.52% within the 3.9–63 μm range (Udden-Wentworth classification). The sample exhibits a poorly sorted distribution with a *D*_v_(50) of 10.6 μm and a span of 2.222 μm, suggesting a relatively broad size distribution. Notably, a fraction (2.73–9.23%) of the material consists of clay-sized particles (<2–3.9 μm), which could be relevant when considering the potential for RCS exposure, as particles <4 μm in size can easily travel deeper into the lungs, reaching the alveoli and leading to potential long-term health issues.^1–4^

**Figure 1:**
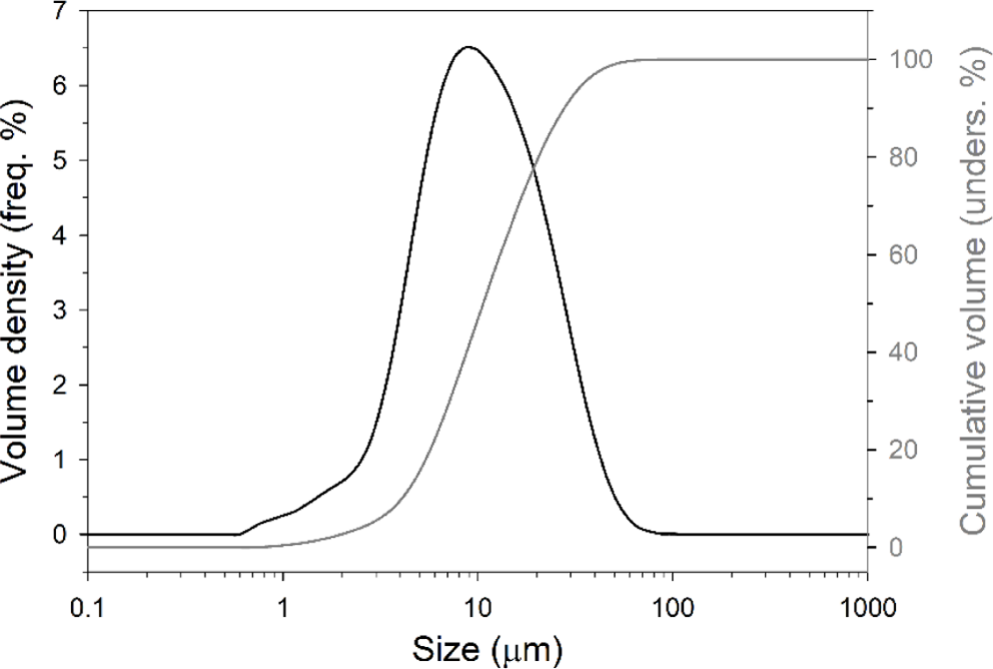
Frequency (black line) and undersize (gray line) size distribution for sample QD.

Chemically, QD contains the silicon and aluminum needed for tobermorite synthesis, but it is relatively poor in calcium and must therefore be supplemented with KRY·AS (M1) or CaO (M2).

### 3.2 Synthesis and Characterization of Tobermorite-Rich Materials

Crystallization test data provide insights into mineralogical phase evolution over time at different temperatures (120, 130, and 140 °C) starting from M1 and M2 mixtures. The numerical values discussed below are given in Table S1.

#### 3.2.1 Synthesis from M1

X-ray powder diffraction (XRPD) patterns of samples obtained after 4 and 8 h show tobermorite-related reflections; however, these are very broad peaks that do not allow for reliable quantitative analysis. At 120 °C (12 h), the amount of tobermorite is 7.2 wt % and gradually increases to 21.9 wt % (144 h) (Figure 2a). At 130 °C (Figure 2b), the initial content is higher (16.2 wt %, 12 h) and remains relatively stable around 18–19 wt % after 24 h. At 140 °C (Figure 2c), the tobermorite reaches a maximum of 26.1 wt % (48 h) before decreasing slightly. Thus, higher temperatures appear to promote faster tobermorite crystallization, peaking earlier at 140 °C. However, prolonged exposure (>108 h) at 140 °C results in a decline, suggesting possible transformation or instability. Brownmillerite, larnite, muscovite, periclase, and, most important for this research, quartz are gradually consumed, the latter more quickly at higher temperatures. Brownmillerite and periclase are no longer observed; it is likely that the former is used as a source of calcium, while the latter becomes amorphous or, as observed at 130 and 140 °C (Figure 2b,c), contributes to the formation of brucite since magnesium does not enter the structure of tobermorite or that of any of the other minerals detected (excluding brucite).^38^

**Figure 2:**
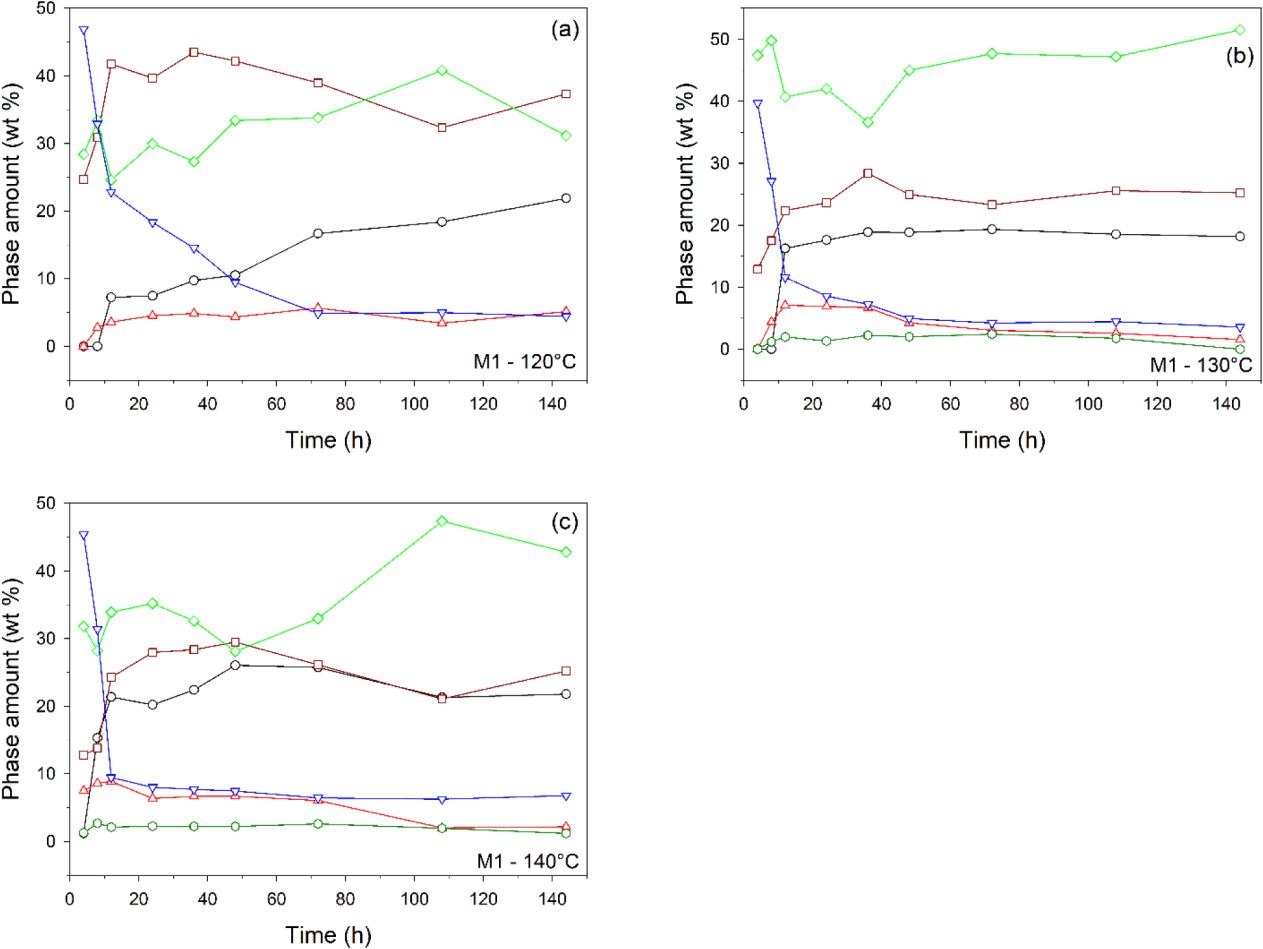
Mineralogical phase composition over time for M1 at 120 (a), 130 (b), and 140 °C (c). For clarity, the error is not reported (in most cases, it falls within the symbol size; see Table S1a for numerical values). Legend: black circle, tobermorite; red triangle up, katoite; brown square, sum of aragonite, calcite, and vaterite; blue triangle down, sum of quartz, muscovite, and larnite; dark green hexagon, brucite; light green diamond, amorphous. Curves with all values equal to zero (Table S1a) are not shown.

The synthesis significantly increases the calcium carbonate content over the source material, including the formation of aragonite, consistent with prior studies.^38^ Calcium carbonate crystallization is likely promoted by the presence of calcite and vaterite in M1 (Table 1) and by interaction with atmospheric carbon dioxide, both inside the autoclave and during subsequent air drying. Furthermore, while calcite exhibits considerable stability across all temperatures, the aragonite content diminishes at 130 and 140 °C, with vaterite no longer detectable at these higher temperatures (Table S1a). It is also observed that katoite, a hydrogarnet with the general formula Ca_3_Al_2_(SiO_4_)_3–*x*_(OH)_4*x*_, where *x* ranges between 1.5 and 3, the latter value in the pure hydroxyl end-member.^104,105^ The crystallization of katoite, a phenomenon documented in other studies concerning tobermorite formation under various conditions,^58,84,85,105–107^ decreases over time, especially at 130 and 140 °C (Figure 2b,c). A considerable amount of amorphous material is also formed. At 120 °C, amorphous material remains relatively high, increasing from 24.6 to 40.8 wt % (Figure 2a); at 130 °C (Figure 2b) the amorphous phase is the major product, reaching 51.5 wt % at 144 h, while at 140 °C (Figure 2c), its amount is lower than that at 130 °C. None of the phases present in ZT are observed in the final products, partly due to the low content (2.5 wt %) of this material used as a catalyst (see Section 2.2).

The reduction of quartz, muscovite, and larnite suggests their consumption in reaction pathways leading to the formation of tobermorite and an amorphous phase. This reaction pathway is particularly evident at 140 °C (Figures 2c and S2). Furthermore, fluctuations in katoite content might point to competitive phase formation.^84,105^ The absence of a clear temporal correlation between the amounts of tobermorite and katoite formed implies that tobermorite does not crystallize directly from katoite but rather through interdependent transformations. The highest amorphous phase fraction at 130 °C indicates incomplete crystallization or phase stabilization issues. This proves that synthesis at 140 °C allows for better crystallization compared to lower temperature values. Overall, after prolonged reaction (>108 h), the amount of crystalline tobermorite decreases, partially transforming into denser, thermodynamically more stable phases like aragonite and, mostly, amorphous.

#### 3.2.2 Synthesis from M2

Even when using the M2 mixture, for syntheses at 120 and 130 °C, tobermorite starts to be well-detected through XRPD after about 12 h (Table S1b). At 120 °C (12 h), the amount is 9.7 wt % and increases steadily until it reaches 38.5 wt % at 144 h (Figure 3a). The initial content at 130 °C (Figure 3b) is much higher (20.8 wt %, 12 h) and reaches 42.6 wt % (144 h). At 140 °C (Figure 3c), the tobermorite increases rapidly from 27.8 wt % (12 h) to 47.8 wt % (144 h). Therefore, as with M1, higher temperatures promote faster tobermorite crystallization (at 140 °C, a concentration of tobermorite not far from the maximum is formed already after 48 h). As with M1, the process leads to the crystallization of calcium carbonates, but here with vaterite occurring also at 130 and 140 °C. The decrease in calcite content at higher temperatures after 108 h does not necessarily reflect dissolution but rather a dynamic balance between dissolution, reprecipitation, and transformation among different carbonate polymorphs (e.g., aragonite). Notably, in M2, portlandite forms through CaO hydration during the initial hours at all investigated temperatures. The subsequent disappearance of this phase after 12 h indicates its complete consumption, thereby contributing to the formation of tobermorite or amorphous material. Katoite was not observed in M2. It is well-established that both katoite and tobermorite crystallize under hydrothermal conditions, provided specific chemical compositions and pH values are maintained.^34,49,64,105^ The experimental conditions employed are expected to favor tobermorite formation. Consequently, its more rapid crystallization may reduce the availability of Ca and Al, thereby inhibiting the nucleation and growth of katoite. The amorphous content is subject to significant variations at all temperatures, with lower values, however, on average at 140 °C (Figure 3c). Significantly, even in M2, tobermorite forms mostly by consuming quartz, a process enhanced at 140 °C as evidenced by the inverse correlation between the two phases (Figure S3). In general, for both M1 and M2, and irrespective of the temperature, Si required for tobermorite crystallization is sourced from quartz (Figure S4).

**Figure 3:**
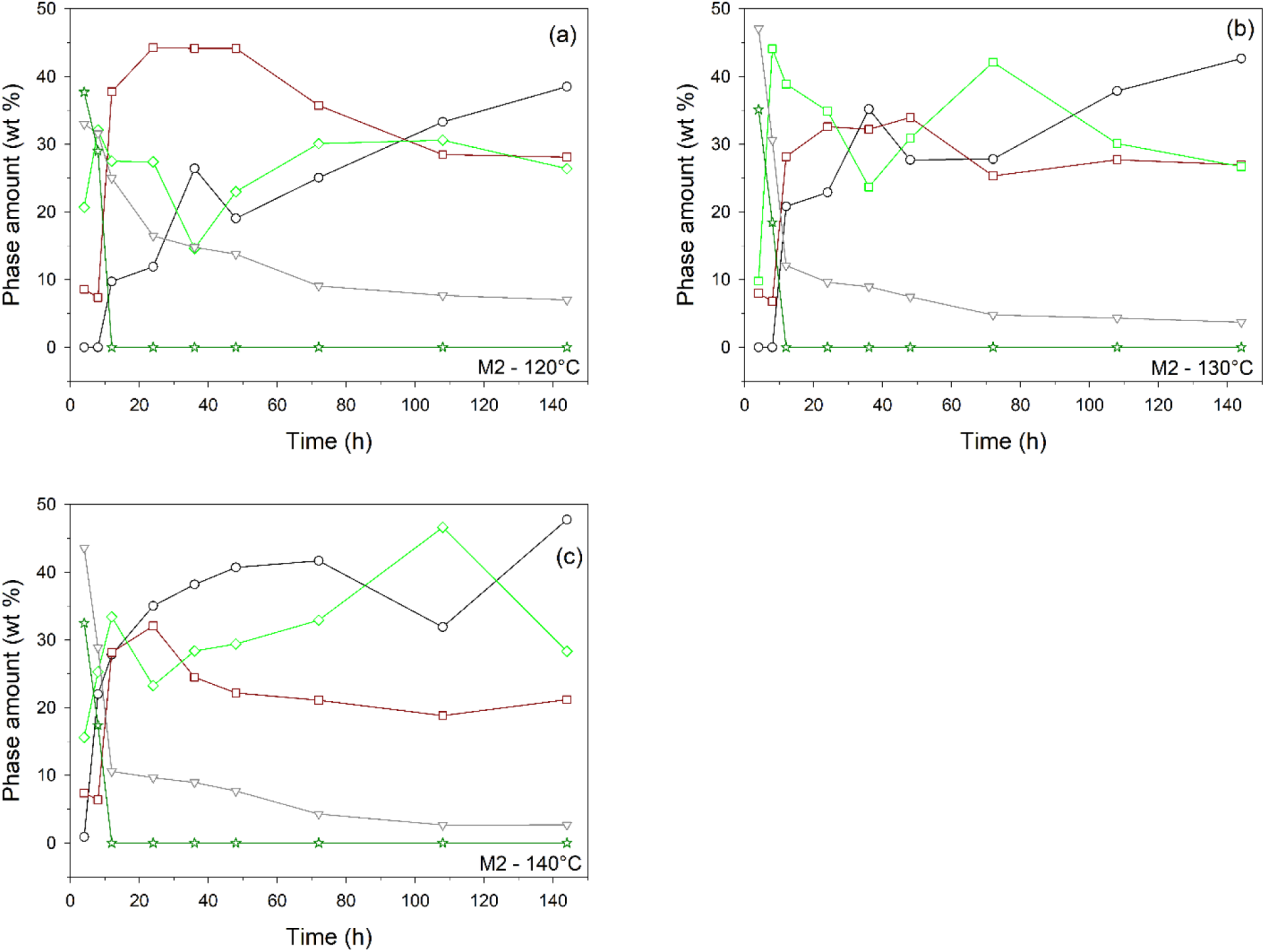
Mineralogical phase composition over time for M2 at 120 (a), 130 (b), and 140 °C (c). For clarity, the error is not reported (in most cases, it falls within the symbol size; see Table S1b for numerical values). Legend: black circle, tobermorite; brown square, sum of aragonite, calcite, and vaterite; gray triangle down, sum of quartz and muscovite; dark green star, portlandite; light green diamond, amorphous. Curves with all values equal to zero (see Table S1b) are not shown.

#### 3.2.3 Crystallization Kinetics of Tobermorite

The α-time plots of the M1 and M2 samples during the isothermal runs 120, 130, and 140 °C, along with the line fits obtained using a first-order reaction model, are shown in Figure 4. In Figure 5, the logarithmic Arrhenius plot for the calculation of the apparent activation energy *E*_a_ from the slope of the regression curve is presented. The data points of the Arrhenius plot represent the kinetic constants *k* calculated from the 120, 130, and 140 °C isothermal runs for samples M1 and M2. The results of the fitting procedure using the first-order kinetic equation are shown in Table 2, including the fitting statistics and the calculated kinetic parameters with their standard errors.

**Table 2:** Results of the Fit Procedure Using a First-Order Reaction Equation for the Isothermal Data of M1 and M2 Samples, including the Calculated Rate Constants, Regression Statistics *R* ^2^, and Activation Energies for the Arrhenius Plot (See Section 3.2.3 for Details)^a^

Sample	Isothermal run 120 (°C)	Isothermal run 130 (°C)	Isothermal run 140 (°C)
**M1**			
*k* (h^–1^)	0.024(3)	0.079(7)	0.11(2)
*R* ^2^	0.928	0.984	0.937
*E*_a_ (kJmol^–1^) = 101(33)			
*R* ^2^ = 0.904			
**M2**			
*k* (h^–1^)	0.017(1)	0.025(5)	0.087(4)
*R* ^2^	0.973	0.843	0.995
*E*_a_ (kJmol^–1^) = 111(34)			
*R* ^2^ = 0.915			

a Errors are reported in parentheses.

**Figure 4:**
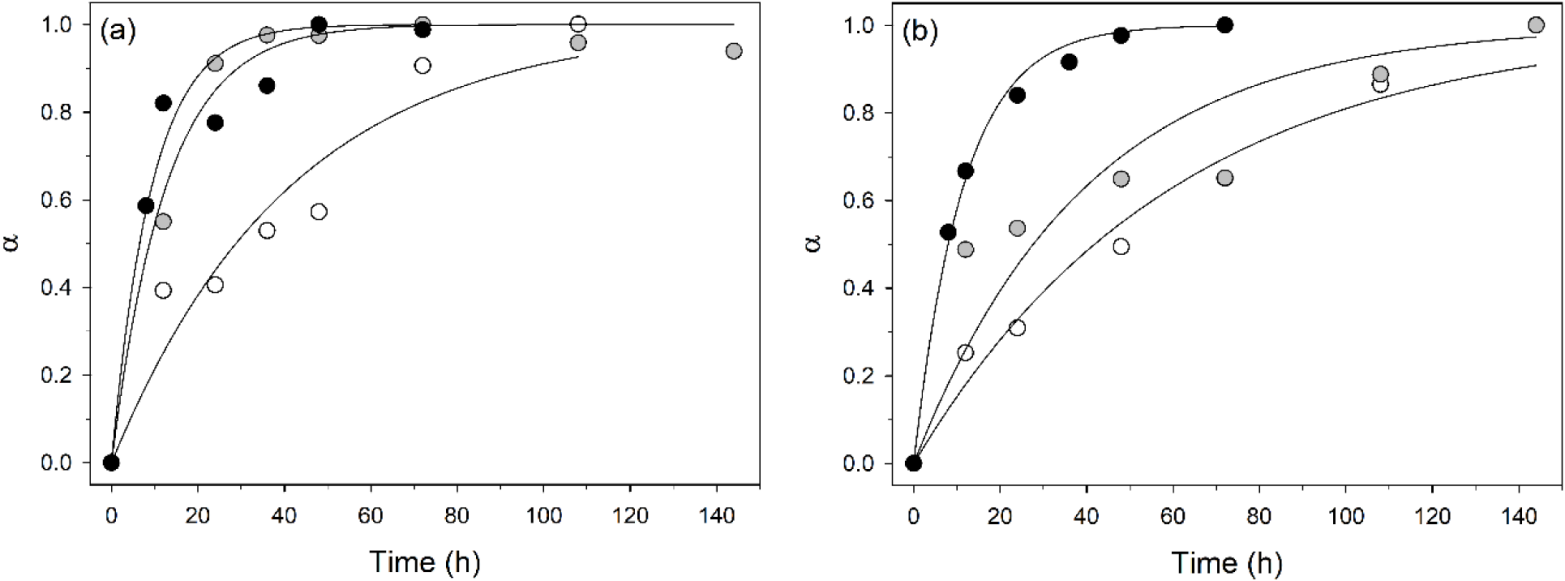
Tobermorite α-time plots of the M1 (a) and M2 (b) mixtures during the isothermal runs 120 (open circle), 130 (gray circle), and 140 °C (black circle). The line fits using a first-order reaction model are also reported.

**Figure 5:**
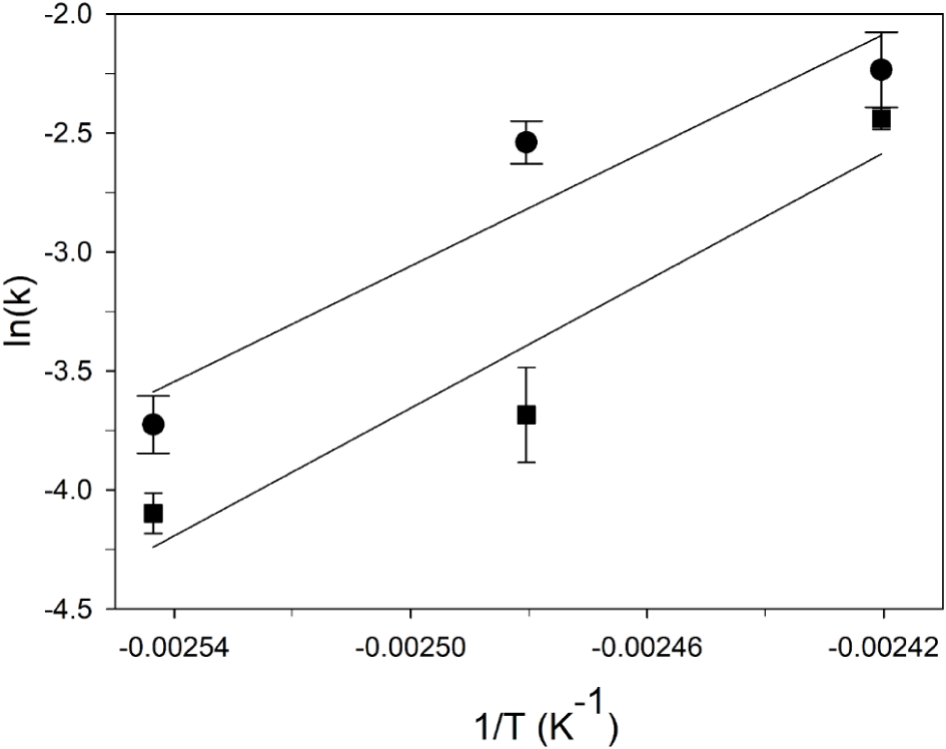
Logarithmic Arrhenius plot for the calculation of *E*_a_ (slope of the regression curve), obtained using kinetic constants *k* calculated from the 120, 130, and 140 °C isothermal runs for the two samples, M1 (circle) and M2 (square).

Although both are first-order reactions, kinetic data for tobermorite crystallization from M1 (primarily composed of larnite, quartz, and a Ca-rich amorphous phase) and M2 (primarily quartz and lime) revealed differences in the reaction products:

-M1 (120 and 130 °C, Figure 2a,b): larnite + quartz + Ca-rich amorphous (likely CSH) → CSH (amorphous to diffraction) + tobermorite;

-M1 (140 °C, Figure 2c): larnite + quartz + Ca-rich amorphous (likely CSH) → tobermorite + carbonates (with inhibition of the formation of amorphous CSH);

-M2 (120, 130, and 140 °C, Figure 3): quartz + lime → CSH (amorphous to diffraction) + tobermorite + carbonates.

With few exceptions, the newly formed phases during tobermorite crystallization are comparable to those observed in previous *in situ* autoclave experiments.^84,108^ These studies investigated the formation of tobermorite from quartz and cement mixtures within the 100–190 °C thermal range. The authors reported that portlandite became unstable and began to decrease after 70 min; quartz diminished at variable rates, and katoite appeared around 40 min. Subsequently, hydroxylellestadite (Ca_10_(SiO_4_)_3_(SO_4_)_3_(OH)_2_) formed, while tobermorite was first observed around 150 min (60 min after reaching 190 °C) and continued to increase until the end of the autoclave process. Therefore, a two-step crystallization process for tobermorite was proposed:^85^

CSH → tobermorite (I)

Quartz, katoite, hydroxylellestadite, (CSH) → Si^4+^, Ca^2+^, Al^3+^, OH^–^ → tobermorite (II)

Reaction (I) is a solid-state transformation, while reaction (II) is a solid–liquid reaction in which tobermorite crystallizes from the liquid phase. These two reactions may proceed interdependently, and in a system with a reactive silica source, the early formation of CSH may slow the dissolution of quartz.^85^

The first-order model used to fit the kinetic curves of the M1 and M2 samples describes a reaction whose rate depends on the concentration of only one reactant. This reactant is thought to be CSH (see the reaction paths described above for the M1 and M2 samples), the precursor of tobermorite. In M1, Ca-rich amorphous phases (CSH) transform into tobermorite, and the crystallization of carbonates appears to inhibit further CSH formation. In M2, the reaction between quartz and lime yields CSH, tobermorite, and carbonates, suggesting that tobermorite likely forms as a secondary product from the CSH phase. Although the reaction pathways observed in this study seem to point to the reaction sequence (I) described by Matsui et al. (2011),^85^ a contribution from the reaction sequence (II) cannot be ruled out, and an interconnection between these two reactions is therefore probable.

Another study^109^ tracked the crystallization of 11 Å-tobermorite from quartz with mean grain sizes of 8 and 16 μm in the 170–210 °C temperature range. This work concluded that the kinetic curves could be described with a slope of either 1 or 0.5, corresponding to an exponent of *n* = 1 (first order) or *n* = 2 (second order), respectively. This is interpreted as mechanisms shifting with the reaction progress from a solution-controlled mechanism to a diffusion-controlled mechanism. The initial step of the reaction is controlled by the dissolution of quartz and its reaction with portlandite, leading to the formation of a layer of CSH surrounding the quartz grains. The second part of the reaction is controlled by the diffusion of SiO_2_ through this layer of CSH-phases; portlandite is expended completely, and the Ca/Si ratio decreases. Tobermorite is then formed by the reaction of quartz with the previously formed CSH. The first stage of the model also applies to the results of this research, as suggested by SEM analysis (Figures S5 and S6) showing the formation of tobermorite by consuming quartz. The second stage of the model, occurring at higher temperatures, cannot be applied to our results.

The calculated apparent activation energies *E*_a_ of 101(33) kJmol^–1^ and 111(34) kJmol^–1^ for samples M1 and M2, respectively (Table 2) are comparable to each other; nevertheless, they are significantly higher than the activation energy (41.54 kJ mol^–1^) determined for the crystallization kinetics of tobermorite at 140–180 °C from sodium silicate solutions (SSS) and synthetic larnite (C_2_S), using an Avrami crystal growth kinetic model.^110^ The difference is likely due to the different experimental conditions (higher temperature range and use of SSS as the precursor). Indeed, the crystallization of tobermorite from quartz, as investigated within the 170–210 °C temperature range in the aforementioned study,^109^ revealed a nonisokinetic reaction (see above) characterized by *E*_a_ values ranging from 16.5 to 33.8 kJ/mol^–1^. This indicates that, even with dissimilar experimental parameters and reagent characteristics, the *E*_a_ values for tobermorite crystallization tend to decrease with increasing temperature.

The larger number of reaction products observed for M1 and, to a lesser extent, M2 compared to the systems described in the literature can be attributed to two main factors: the increased complexity of the starting materials (especially M1) and the dynamic synthesis method (performed on a tilting table; see Section 2.2). Despite the higher activation energy required, this nonstatic approach appears to favor substituted tobermorite synthesis at a relatively lower temperature than other systems documented in the literature,^109,110^ preventing the formation of competing phases. Nevertheless, 120 °C seems to be a minimum threshold temperature for tobermorite formation, as 144 h tests conducted at 110 °C yielded no tobermorite (or even katoite) and left a considerable amount of residual quartz (see, for example, Figure S7).

### 3.3 Thermal Behavior

The thermogravimetric and its first derivative analysis (TGA-DTG), thermodifferential analysis (DTA), and mass spectrometry of evolved gas (MSEGA) of the materials with the highest tobermorite content (i.e., M1-48H-140 and M2-144H-140) are shown in Figures 6 and 7, respectively.

**Figure 6:**
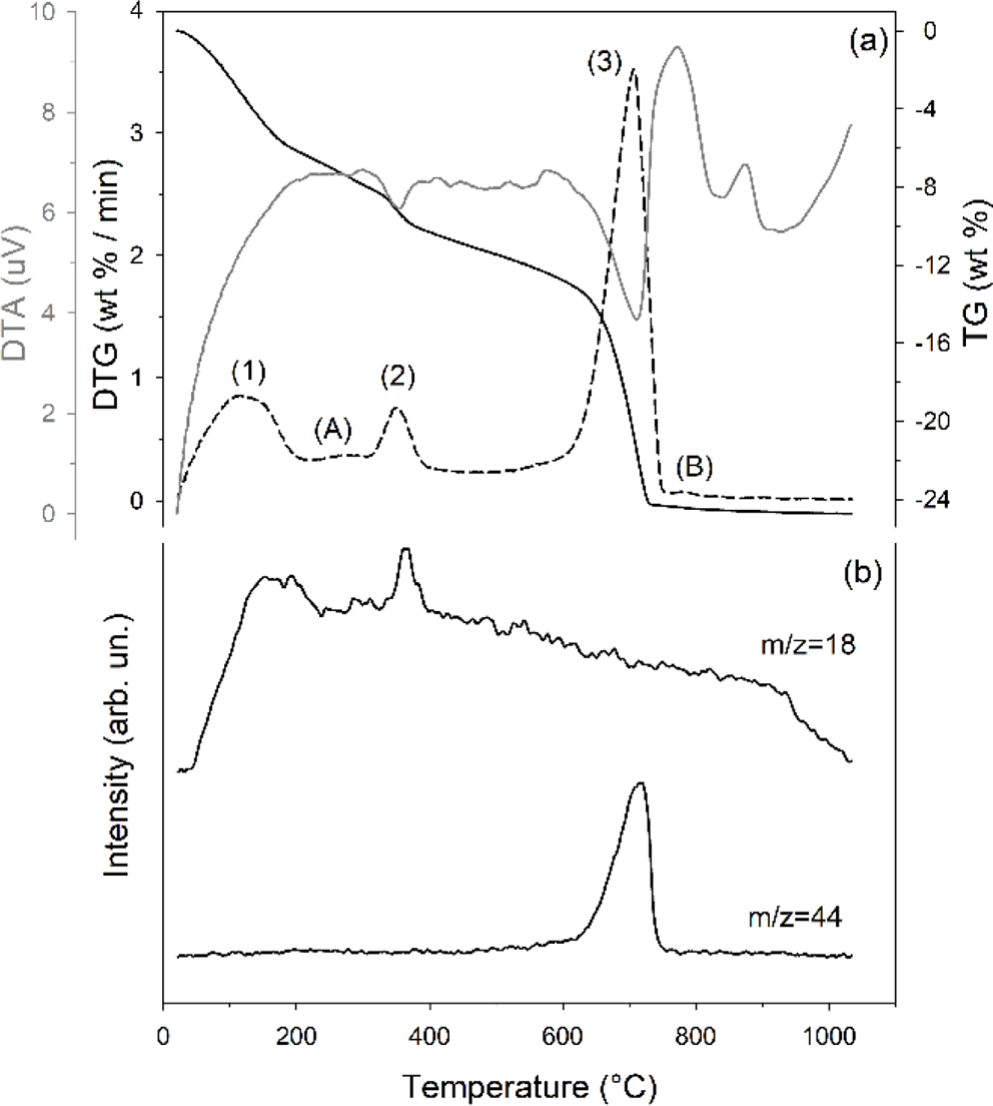
Thermal behavior of samples M1-48H-140. (a) TGA (black solid lines), DTG (black dashed lines), and DTA (gray line) curves (the maxima on the DTA curve denote exothermic reactions); (b) MSEGA curves for H_2_O (*m*/*z* = 18) and CO_2_ (*m*/*z* = 44).

**Figure 7:**
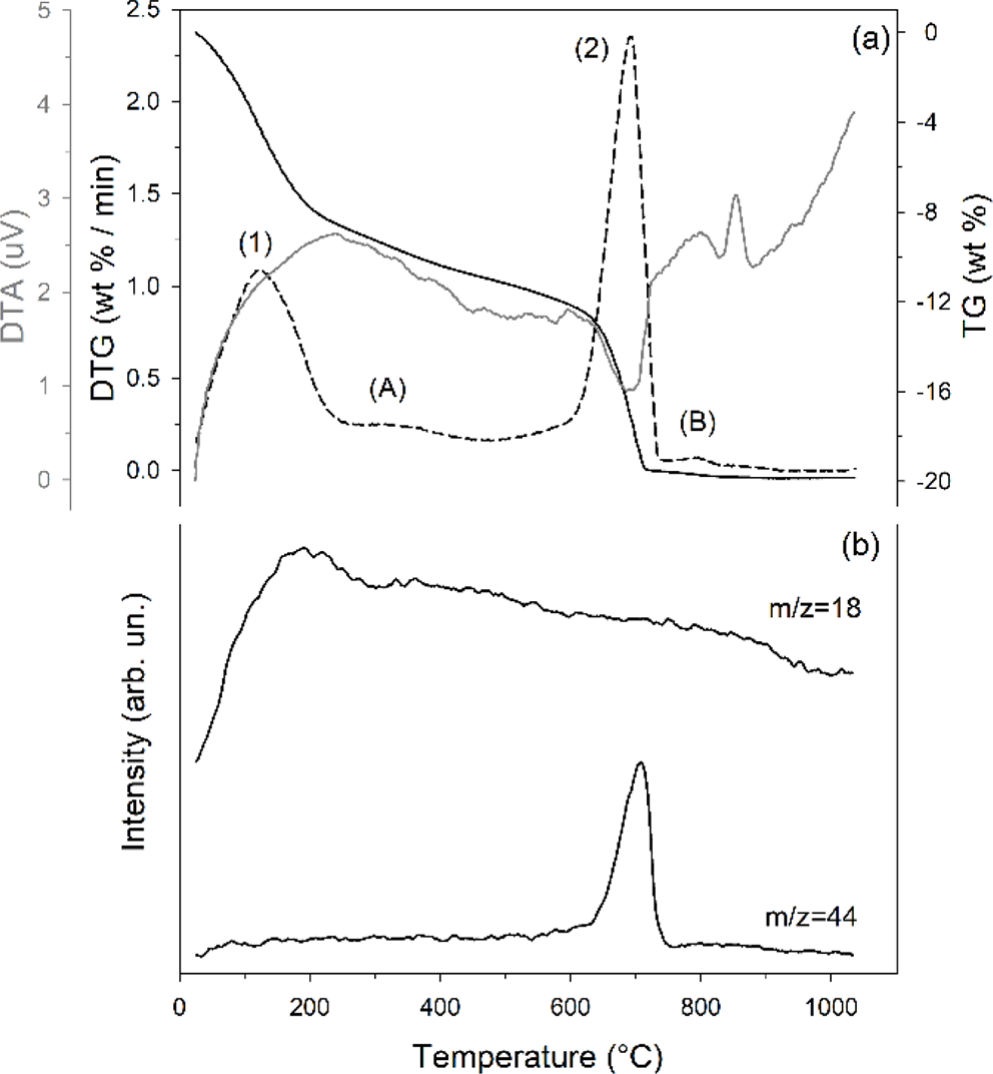
Thermal behavior of samples M2-144H-140. TGA/DTG/DTA (a) and MSEGA (b) curves. Legend like that in Figure 6.

Sample M1-48H-140 (Figure 6a) shows three primary thermal events: (1) 25–215 °C (maximum reaction rate at 115 °C; mass loss of 6.40 wt %), (2) 305–410 °C (maximum at 351 °C; mass loss of 2.31 wt %), and (3) 507–763 °C (maximum at 706 °C; mass loss of 12.84 wt %). Two additional minor reactions (A and B, respectively) occur between 215 and 305 °C (maximum not defined; mass loss of 1.74 wt %) and between 770 and 821 °C, peaking at 786 °C, with a mass loss of 0.13 wt %. Reaction (1), evidenced by the release of water (*m*/*z* = 18, Figure 6b), corresponds to the removal of zeolitic water from tobermorite. Reaction (2), also coupled with water release, is attributed to the simultaneous dehydroxylation of katoite,^111^ brucite,^112,113^ and other amorphous hydroxides or the dehydration of amorphous CSH. Reaction (3), as indicated by the release of CO_2_ (*m*/*z* = 44, Figure 6b), results from the decarbonation of calcite; a minor contribution to this reaction is from aragonite, suggested by the small shoulder in the DTG curve at about 590 °C, consistent with the QPA showing an aragonite content of 4.7 wt % (Table S1a). The overall mass loss of reaction (3) allows the calculation of the amount of calcium carbonates, which is 29.2 wt%, in agreement with QPA (29.5 wt %, sum of calcite and aragonite). The minor reaction (A) is attributed to the final thermal decomposition of amorphous hydroxides or the dehydration of amorphous CSH.^111^ Reaction (B), also observed in sample M2-144H-140 and consistent with prior research,^38^ is more difficult to interpret. This difficulty arises because the small amount of material involved in the reaction prevents the detection of any released gases. Beyond the insights provided by TGA, the DTA curve (Figure 6a) reveals several exothermic effects above 800 °C, indicative of the formation of high-temperature phases such as wollastonite.^111^

Sample M2-144H-140 shows two main thermal events (Figure 7a): (1) 25–265 °C (maximum reaction rate at 126 °C; mass loss of 8.84 wt %) and (2) 510–755 °C (maximum at 695 °C; mass loss of 8.41 wt %). As in M1-48H-140, two additional minor reactions (A and B, respectively) occur between 265 and 400 °C (maximum not defined; mass loss of 1.54 wt %) and between 765 and 855 °C, peaking at 805 °C, with a mass loss of 0.20 wt %.

Thermal events (1) and (2) proceed via reaction mechanisms analogous to those previously observed for the preceding sample, involving dehydration and decarbonation, respectively. Evidence of these mechanisms is also provided by evolved gas analyses (Figure 7b). From the mass change associated with the decarbonation reaction, a total carbonate content (calcite + aragonite) of 19.1 wt % was calculated. This aligns well with the XRPD data (21.2 wt %, Table S1b). Notably, the reaction corresponding to the dehydroxylation of katoite and brucite (reaction (2) in M1-48-140) was not observed in sample M2-144-140, consistent with the absence of these phases (Table S1b). Similar to M1-48H-140, the DTA curve (Figure 7a) exhibits prominent exothermic effects above 800 °C, corresponding to the formation of high-temperature phases.

A significant observation for both samples is the lack of dehydroxylation reactions associated with tobermorite. This indicates that the charge compensation required for Al^3+^ for Si^4+^ substitutions occurs through Ca^2+^ entry into the interlayer, rather than by (OH)^−^ for O^2–^ substitutions within the 7-fold Ca–O polyhedron. In fact, at low temperatures (120–140 °C), the kinetics of hydrolysis and deprotonation are slower. Consequently, even with a high (OH)^−^ concentration (synthesis occurs in an alkaline environment), the available energy is not sufficient to favor the incorporation of hydroxyls into crystalline frameworks, particularly where (OH)^−^ should occupy structural positions, e.g., bridging Si–OH–Al.^114,115^ Therefore, at lower temperatures, it is both thermodynamically and kinetically more favorable to incorporate Ca^2+^ into interlayer positions than to build hydroxylated sheets into the silicate layer, a process conversely favored at higher temperatures.^116^ Moreover, the incorporation of hydroxyl into solid phases requires specific local bonding geometries (e.g., bridging OH, or terminal OH). At lower temperatures, the mobility of structural water and framework rearrangements is limited. Hence, nonhydroxylated but hydrated phases (e.g., Ca-rich CSH amorphous material and tobermorite with extra interlayer Ca^2+^) are more likely to form.^87,116^ Therefore, Ca^2+^ in interlayer sites tends to minimize formation energy relative to many substituted cations, meaning that the system is more thermodynamically stable when Ca^2+^ is present. In fact, interlayer Ca^2+^ helps preserve structural features such as basal spacing and prevents undesirable rearrangements and substitution by alkali or transition metal cations, although it increases exchangeability and decreases structural stability.^38,45,115,117^

### 3.4 Indications from the SEM Investigation

Quartz is an easily detectable mineral using XRPD, and the present Italian normative (UNI ISO 24095:2022) indicates XRPD as the analytical technique to use for crystalline silica detection (in addition to grain size analyses); however, low amounts (usually less than about 0.5 wt %) could produce diffraction signals that cannot be discriminated from the background. SEM images performed on samples M1-48H-140 and M2-144H-140 (Figures S8 and S9, respectively) support the XRPD results, indicating the absence of quartz. Complementary EDX maps reveal no discrete silica-rich grains, thereby suggesting that quartz is either absent or negligible following the synthesis procedures.

## 4 Conclusions

Through this research, the formation of tobermorite was investigated using waste from quartzite processing containing RCS as the source of silica.

In synthetic systems like this, higher temperatures promote rapid dissolution of quartz, providing silica for tobermorite and, in M1, katoite. Indeed, quartz is completely consumed at 130 and 140 °C but persists longer at 120 °C. However, although higher temperatures can enhance the initial nucleation rates of tobermorite, extended exposure or extreme conditions can destabilize its crystal structure, thus impairing its overall growth, particularly when employing precursors with more complex compositions (M1).

For the M1 composition, synthesis at 140 °C appears most effective for tobermorite formation, achieving the peak content more rapidly. Conversely, 120 °C leads to steady but slower crystallization with persistent amorphous material, while 130 °C retains the highest percentage of the amorphous phase, suggesting incomplete crystallization. Similarly, for M2, higher temperatures promote faster tobermorite crystallization; at 140 °C, nearly maximum formation occurs earlier (48 h), whereas at lower temperatures, the increase is more gradual. Therefore, under hydrothermal alkaline conditions, quartz grains gradually dissolve, releasing reactive silica that combines with calcium supplied by CaO or KRY·AS to form amorphous CSH. These amorphous phases act as precursors for the nucleation and growth of tobermorite, which progressively consumes quartz. During crystallization, competing phases may form, including katoite, metastable carbonate polymorphs (calcite, aragonite, vaterite) due to CO_2_ uptake, and persistent amorphous CSH at lower temperatures.

Beyond considerations of crystallization kinetics, the combined XRPD and SEM data confirm the successful recovery of the QD, with RCS transformed into safe phases. This unequivocally demonstrates that the outlined process fulfills the criteria for an End-of-Waste designation

Furthermore, this study demonstrates that the synthesis process involves the crystallization of calcium carbonates, highlighting a promising direction for future research on CO_2_ storage.

## Supplementary Material





## Notes

The Supporting Information file (SupportingInformation.pdf) offers a detailed description of the instrument, analytical methods, and experimental conditions used; this file also includes selected SEM images of the QD sample (Figure S1), correlations between the amounts of various phases formed/consumed during synthesis (Figures S2–S4), SEM and SEM-EDX images/analyses of selected synthesis products (Figures S5–S6, S8–S9), XRPD pattern of a synthesis test at 110 °C (Figure S7), and a table presenting the quantitative mineralogical composition for each synthesis step (Table S1) (PDF)
Appendix A: XRPD patterns of QD, KRY·AS, ZT, M1, M2 and samples reported in Table S1 (ZIP)

## Abbreviations

RCS: Respirable crystalline silica
CSH: calcium silicate hydrate
QPA: quantitative phase analyses
SEM: scanning electron microscopy
XRPD: X-ray powder diffraction
TGA: thermogravimetric analyses
DTA: thermodifferential analyses
MSEGA: mass spectrometry of evolved gases analyses
QD: quartzite dust
ZT: zeolitic tuff
M1: mixture prepared by dosing 75, 22.5, and 2.5 wt % of KRY·AS, QD, and ZT, respectively
M2: mixture prepared by dosing 42.0, 55.5, and 2.5 wt % of CaO, QD, and ZT

## References

[1] Murugadoss S., Lison D., Godderis L., Van Den Brule S., Mast J., Brassinne F., Sebaihi N., Hoet P. H. (2017). Toxicology
of Silica Nanoparticles: An Update. Arch. Toxicol..

[2] Sato T., Shimosato T., Klinman D. M. (2018). Silicosis and Lung Cancer: Current
Perspectives. Lung Cancer Targets Ther..

[3] Hoy R. F., Jeebhay M. F., Cavalin C., Chen W., Cohen R. A., Fireman E., Go L. H. T., León-Jiménez A., Menéndez-Navarro A., Ribeiro M., Rosental P. (2022). Current Global
Perspectives on Silicosis—Convergence of Old and Newly Emergent
Hazards. Respirology.

[4] Liu J. Y., Sayes C. M. (2022). A Toxicological
Profile of Silica Nanoparticles. Toxicol. Res..

[5] OSHA OSHA 1926.1153. https://www.osha.gov/laws/regs/regulations/standardnumber/1926/1926.1153 (accessed 29 April 2025).

[6] 44/2020/IT. https://www.normattiva.it/uri-res/N2Ls?urn:nir:stato:decreto.legislativo:2020;44 (accessed 29 April 2025).

[7] 2398/2017/CE. http://data.europa.eu/eli/dir/2017/2398/oj/ita (accessed 29 April 2025).

[8] Leso V., Fontana L., Romano R., Gervetti P., Iavicoli I. (2019). Artificial
Stone Associated Silicosis: A Systematic Review. Int. J. Environ. Res. Public Health.

[9] Saka M. B., Hashim M. H. B. M. (2024). Critical Assessment of the Effectiveness of Different
Dust Control Measures in a Granite Quarry. J.
Public Health Policy.

[10] Wood C., Yates D. (2020). Respiratory Surveillance
in Mineral Dust-Exposed Workers. Breathe.

[11] Anlimah F., Gopaldasani V., MacPhail C., Davies B. (2023). A Systematic Review
of the Effectiveness of Dust Control Measures Adopted to Reduce Workplace
Exposure. Environ. Sci. Pollut. Res..

[12] Peruzzi C. P., Brucker N., Bubols G., Cestonaro L., Moreira R., Domingues D., Arbo M., Olivo Neto P., Knorst M. M., Garcia S. C. (2022). Occupational Exposure to Crystalline
Silica and Peripheral Biomarkers: An Update. J. Appl. Toxicol..

[13] Directive 2008/98/EC, 2008; Vol. 312. http://data.europa.eu/eli/dir/2008/98/oj/eng (accessed 20 May 2025).

[14] Pongrácz E., Pohjola V. J. (2004). Re-Defining
Waste, the Concept of Ownership and the
Role of Waste Management. Resour., Conserv.
Recycl..

[15] Latunussa C. E. L., Ardente F., Blengini G. A., Mancini L. (2016). Life Cycle
Assessment
of an Innovative Recycling Process for Crystalline Silicon Photovoltaic
Panels. Sol. Energy Mater. Sol. Cells.

[16] Assi A., Bilo F., Zanoletti A., Ponti J., Valsesia A., La Spina R., Zacco A., Bontempi E. (2020). Zero-Waste Approach
in Municipal Solid Waste Incineration: Reuse of Bottom Ash to Stabilize
Fly Ash. J. Cleaner Prod..

[17] Cho B. H., Nam B. H., An J., Youn H. (2020). Municipal
Solid Waste
Incineration (MSWI) Ashes as Construction Materials-A Review. Mater. Basel Switz..

[18] Hicks J., Yager J. (2006). Airborne Crystalline Silica Concentrations at Coal-Fired Power Plants
Associated with Coal Fly Ash. J. Occup. Environ.
Hyg..

[19] Jena S. K., Dash N., Rath S. S. (2021). A Novel Application
of Waste Cement
Clinker Dust in the Extraction of Potash from Mica Scraps. Resour., Conserv. Recycl..

[20] Adazabra A. N., Viruthagiri G., Atingabono J. (2023). Developing Fired Clay Bricks by Incorporating
Scrap Incinerated Waste and River Dredged Sediment. Process Saf. Environ. Prot..

[21] Beddaa H., Tchiotsop J., Ben Fraj A., Somé C. (2023). Reuse of River
Sediments in Pervious Concrete: Towards an Adaptation of Concrete
to the Circular Economy and Climate Change Challenges. Constr. Build. Mater..

[22] Beddaa H., Fraj A. B., Ducléroir S. (2021). Experimental Study on River Sediment
Incorporation in Concrete as a Full Aggregate Replacement: Technical
Feasibility and Economic Viability. Constr.
Build. Mater..

[23] Ducman V., Bizjak K. F., Likar B., Kolar M., Robba A., Imperl J., Božič M., Gregorc B. (2022). Evaluation of Sediments
from the River Drava and Their Potential for Further Use in the Building
Sector. Materials.

[24] Hussain M., Levacher D., Leblanc N., Zmamou H., Djeran-Maigre I., Razakamanantsoa A., Saouti L. (2022). Reuse of Harbour and River Dredged
Sediments in Adobe Bricks. Clean. Mater..

[25] Singh P., Vitone C., Baudet B. A., Cotecchia F., Notarnicola M., Plötze M., Puzrin A. M., Goli V. S. N. S., Mali M., Petti R. (2025). Characterisation Remediation
and Valorisation of Contaminated Sediments: A Critical Review. Environ. Geotech..

[26] Zhang K., Wei Q., Jiang S., Shen Z., Zhang Y., Tang R., Yang A., Chow W. K. C. (2022). Utilization of Dredged River Sediment
in Preparing Autoclaved Aerated Concrete Blocks. J. Renew. Mater..

[27] Bernardin, A. M. Recycling and Reuse of Bottom Ashes from Municipal Solid-Waste Incineration Plants in Building Materials. In Advances in the Toxicity of Construction and Building Materials. Pacheco-Torgal, F. ; Falkinham, J. O. ; Gałaj, J. A. , Woodhead Publishing Series in Civil and Structural Engineering; Woodhead Publishing, 2022, 285–298. 10.1016/B978-0-12-824533-0.00001-3.

[28] Chen T., Duan L., Cheng S., Jiang S., Yan B. (2023). The Preparation
of Paddy Soil Amendment Using Granite and Marble Waste: Performance
and Mechanisms. J. Environ. Sci..

[29] Lehmusto J., Tesfaye F., Karlström O., Hupa L. (2024). Ashes from Challenging
Fuels in the Circular Economy. Waste Manage..

[30] López-Uceda A., Cantador-Fernández D., Da Silva P. R., de Brito J., Fernández-Rodríguez J. M., Jiménez J. R. (2024). Mechanical
and Durability Performance of Self-Compacting Mortars Made with Different
Industrial by-Products as Fillers. Constr. Build.
Mater..

[31] Smalakys G. (2020). The Hydrothermal
Synthesis of 1.13 Nm Tobermorite from Granite Sawing Powder Waste. Ceram. Silik..

[32] Siauciunas R., Smalakys G., Dambrauskas T. (2021). Porosity of Calcium Silicate Hydrates
Synthesized from Natural Rocks. Materials.

[33] Coleman N. J. (2006). Interactions
of Cd­(II) with Waste-Derived 11 Å Tobermorites. Sep. Purif. Technol..

[34] Coleman N. J. (2005). Synthesis
Structure and Ion Exchange Properties of 11Å Tobermorites from
Newsprint Recycling Residue. Mater. Res. Bull..

[35] Guo X., Li D. (2019). Solidification/Adsorption of Heavy Metals by FA/FA-MSWI Based Al-Substituted
Tobermorite. J. Wuhan Univ. Technol., Mater.
Sci. Ed..

[36] Komarneni S. (1985). Heavy Metal
Removal from Aqueous Solutions by Tobermorites and Zeolites. Nucl. Chem. Waste Manage..

[37] Komarneri S., Roy D. M., Roy R. (1982). Al-Substituted
Tobermorite: Shows
Cation Exchange. Cem. Concr. Res..

[38] Malferrari D., Bernini F., Di Giuseppe D., Scognamiglio V., Gualtieri A. F. (2022). Al-Substituted Tobermorites: An Effective
Cation Exchanger
Synthesized from “End-of-Waste” Materials. ACS Omega.

[39] Qi, H. ; Du, H. ; Liang, S.-P. ; Yin, C. Study On Adsorption Of Pb2+ By Synthetic Tobermorite. In International Conference on Mechanic Automation and Control Engineering; IEEE, 2010; pp. 1912–1917. DOI: 10.1109/MACE.2010.5536601.

[40] Tsutsumi T., Nishimoto S., Kameshima Y., Miyake M. (2014). Hydrothermal Preparation
of Tobermorite from Blast Furnace Slag for Cs^+^ and Sr^2+^ Sorption. J. Hazard. Mater..

[41] Zou J., Guo C., Zhou X., Sun Y., Yang Z. (2018). Sorption Capacity and
Mechanism of Cr3+ on Tobermorite Derived from Fly Ash Acid Residue
and Carbide Slag. Colloids Surf., A.

[42] Xing L., Li X., Cao P., Luo J., Jiang H. (2024). Stepwise Extraction
and Utilization of Silica and Alumina from Coal Fly Ash by Mild Hydrothermal
Process. Process Saf. Environ. Prot..

[43] Yang J., Sun H., Peng T., Zeng L., Zhou X. (2022). Mild Hydrothermal Synthesis
of 11Å-TA from Alumina Extracted Coal Fly Ash and Its Application
in Water Adsorption of Heavy Metal Ions (Cu­(II) and Pb­(II)). Int. J. Environ. Res. Public Health.

[44] Bonaccorsi E., Merlino S. (2018). Modular Microporous
Minerals: Cancrinite-Davyne Group
and C-S-H Phases. Rev. Mineral Geochem..

[45] Merlino S., Bonaccorsi E., Armbruster T. (2001). The Real Structure
of Tobermorite
11A: Normal and Anomalous Forms, OD Character and Polytypic Modifications. Eur. J. Mineral..

[46] Biagioni C., Merlino S., Bonaccorsi E. (2015). The Tobermorite
Supergroup: A New
Nomenclature. Mineral. Mag..

[47] Pekov I. V., Zubkova N. V., Chukanov N. V., Merlino S., Yapaskurt V. O., Belakovskiy D. I., Loskutov A. B., Novgorodova E. A., Vozchikova S. A., Britvin S. N., Pushcharovsky D. Y. (2022). Paratobermorite
Ca4­(Al0.5Si0.5)­2Si4O16­(OH)·2H2O·(Ca·3H_2_O),
a New Tobermorite-Supergroup Mineral with a Novel Topological Type
of the Microporous Crystal Structure. Am. Mineral..

[48] McConnell J. D. C. (1954). The
Hydrated Calcium Silicates Riversideite, Tobermorite, and Plombierite. Mineral. Mag. J. Mineral. Soc..

[49] Diamond S., White J. L., Dolch W. L. (1966). Effects
of Isomorphous Substitution
in Hydrothermally-Synthesized Tobermorite. Am.
Mineral..

[50] Galvánková L., Másilko J., Solný T., Štěpánková E. (2016). Tobermorite
Synthesis Under Hydrothermal Conditions. Procedia
Eng..

[51] Liao W., Li W., Fang Z., Lu C., Xu Z (2019). Effect of Different
Aluminum Substitution Rates on the Structure of Tobermorite. Materials.

[52] Chiang Y. W., Ghyselbrecht K., Santos R. M., Meesschaert B., Martens J. A. (2012). Synthesis of Zeolitic-Type
Adsorbent Material from
Municipal Solid Waste Incinerator Bottom Ash and Its Application in
Heavy Metal Adsorption. Catal. Today.

[53] Ding J., Tang Z., Ma S., Wang Y., Zheng S., Zhang Y., Shen S., Xie Z. (2016). A Novel Process for
Synthesis of Tobermorite Fiber from High-Alumina Fly Ash. Cem. Concr. Compos..

[54] Hou X., Ma S., Wang X., Liu R., Ibrahim M. (2024). Hydrothermal Transformation
of Fly Ash to Tobermorite or Katoite: Impact of Ca and Si Concentration
in the Liquid Phase without Alkali Activation. Ceram. Int..

[55] Hou X., Ma S., Wang X., Ou Y., Liu R. (2023). Effects of Alkali Activation
and Hydrothermal Processes on the Transformation of Fly Ash into Al-Substituted
Tobermorite Fiber. Constr. Build. Mater..

[56] Ma W., Brown P. W. (1997). Hydrothermal Synthesis
of Tobermorite from Fly Ashes. Adv. Cem. Res..

[57] Wajima T. (2016). Synthesis
of Tobermorite from the Ash after Treatment of Asbesto-Containing
Disaster Waste, and Its Removal Ability of Cs­(I) from Aqueous Solution. Eng. J..

[58] Coleman N. J., Brassington D. S. (2003). Synthesis of Al-Substituted 11 Å Tobermorite from
Newsprint Recycling Residue: A Feasibility Study. Mater. Res. Bull..

[59] Hurt A. P., Coleman A. A., Ma H., Li Q., Coleman N. J. (2022). Calcium
Silicate Hydrate Cation-Exchanger from Paper Recycling Ash and Waste
Container Glass. Ceramics.

[60] Coleman N. J., Li Q., Raza A. (2014). Synthesis
Structure and Performance of Calcium Silicate
Ion Exchangers from Recycled Container Glass. Physicochem. Probl. Miner. Process..

[61] Lamidi Y. D., Owoeye S. S., Abegunde S. M. (2020). Preparation and Characterization
of Synthetic Tobermorite (CaO–Al_2_O_3_–SiO_2_–H_2_O) Using Bio and Municipal Solid Wastes
as Precursors by Solid State Reaction. Bol.
Soc. Esp. Cerámica Vidr..

[62] Reinik J., Heinmaa I., Mikkola J.-P., Kirso U. (2007). Hydrothermal Alkaline
Treatment of Oil Shale Ash for Synthesis of Tobermorites. Fuel.

[63] Smalakys G., Siauciunas R. (2018). The Synthesis of 1.13 Nm Tobermorite
from Carbonated
Opoka. J. Therm. Anal. Calorim..

[64] Coleman N. J., Trice C. J., Nicholson J. W. (2009). 11 Å Tobermorite from Cement
Bypass Dust and Waste Container Glass: A Feasibility Study. Int. J. Miner. Process..

[65] Jing Z., Jin F., Hashida T., Yamasaki N., Ishida H. (2007). Hydrothermal Solidification
of Blast Furnace Slag by Formation of Tobermorite. J. Mater. Sci..

[66] Saldia S., Bacosa H., Vegafria M. C., Zoleta J., Hiroyoshi N., Empig E., Calleno C., Cantong W., Ibarra E., Aguilos M., Amparado R. (2024). Combined Potential
of Quarry Waste
Fines and Eggshells for the Hydrothermal Synthesis of Tobermorite
at Varying Cement Content. Sustainability.

[67] Gualtieri A. F., Cavenati C., Zanatto I., Meloni M., Elmi G., Gualtieri M. L. (2008). The Transformation
Sequence of Cement–Asbestos
Slates up to 1200°C and Safe Recycling of the Reaction Product
in Stoneware Tile Mixtures. J. Hazard. Mater..

[68] Pugnaloni A., Lucarini G., Rubini C., Smorlesi A., Tomasetti M., Strafella E., Armeni T., Gualtieri A. F. (2015). Raw and
Thermally Treated Cement Asbestos Exerts Different Cytotoxicity Effects
on A549 Cells in Vitro. Acta Histochem..

[69] Viani A., Gualtieri A. F., Pollastri S., Rinaudo C., Croce A., Urso G. (2013). Crystal Chemistry of the High Temperature Product of Transformation
of Cement-Asbestos. J. Hazard. Mater..

[70] Gualtieri A. F., Gualtieri M. L., Tonelli M. (2008). In Situ ESEM Study of the Thermal
Decomposition of Chrysotile Asbestos in View of Safe Recycling of
the Transformation Product. J. Hazard. Mater..

[71] Ligabue M. L., Gualtieri A. F., Gualtieri M. L., Malferrari D., Lusvardi G. (2020). Recycling of Thermally
Treated Cement-Asbestos for
the Production of Porcelain Stoneware Slabs. J. Cleaner Prod..

[72] Ligabue M. L., Saburit A., Lusvardi G., Malferrari D., Garcia-Ten J., Monfort E. (2022). Innovative Use of Thermally
Treated
Cement-Asbestos in the Production of Foaming Materials: Effect of
Composition, Foaming Agent, Temperature and Reaction Time. Constr. Build. Mater..

[73] Komarneni S., Komarneni J. S., Newalkar B., Stout S. (2002). Microwave-Hydrothermal
Synthesis of Al-Substituted Tobermorite from Zeolites. Mater. Res. Bull..

[74] Lothenbach B., Jansen D., Yan Y., Schreiner J. (2022). Solubility
and Characterization of Synthesized 11 Å Al-Tobermorite. Cem. Concr. Res..

[75] Liu X., Hao X., Wang C., Su M., Song J., Lou Z., Zhang M. (2025). Role of Ca/Si Ratio in Carbonate-Induced Tobermorite Corrosion: Insights
from First-Principles Simulations. Mater. Des..

[76] Siauciunas R., Steponaityte L., Dzvinka M., Kareiva A. (2025). Influence of Al2O3
Additive on the Synthesis Kinetics of 1.13 Nm Tobermorite, and Its
Crystallinity and Morphology. Materials.

[77] Kikuma J., Tsunashima M., Ishikawa T., Matsuno S., Ogawa A., Matsui K., Sato M. (2011). Effects of Quartz Particle Size and
Water-to-Solid Ratio on Hydrothermal Synthesis of Tobermorite Studied
by in-Situ Time-Resolved X-Ray Diffraction. J. Solid State Chem..

[78] Corro-Escorcia I. A., Hernández-Ávila J., Cerecedo-Sáenz E., Barrientos-Hernández F. R., Cruz-Hernández M., Toro N., Gálvez E., Gutiérrez-Amador M. P., Salinas-Rodríguez E. (2025). Synthesis
of Tobermorite 11 Å
during the Formation of Autoclaved Aerated Concrete with the Addition
of Diatomite. Results Mater..

[79] Black L., Garbev K., Stumm A. (2009). Structure Bonding and
Morphology
of Hydrothermally Synthesised Xonotlite. Adv.
Appl. Ceram..

[80] Chan C. F., Sakiyama M., Mitsuda T. (1978). Kinetics of the CaO-Quartz-H_2_O Reaction at 120° to 180°C in Suspensions. Cem. Concr. Res..

[81] El-Hemaly S. A. S., Mitsuda T., Taylor H. F. W. (1977). Synthesis
of Normal and Anomalous
Tobermorites. Cem. Concr. Res..

[82] Gabrovšek R., Kurbus B., Mueller D., Wieker W. (1993). Tobermorite Formation
in the System CaO, C_3_S-SiO_2_-Al_2_O_3_-NaOH-H_2_O under Hydrothermal Conditions. Cem. Concr. Res..

[83] Guo X., Meng F., Shi H. (2017). Microstructure
and Characterization
of Hydrothermal Synthesis of Al-Substituted Tobermorite. Constr. Build. Mater..

[84] Kikuma J., Tsunashima M., Ishikawa T., Matsuno S., Ogawa A., Matsui K., Sato M. (2010). *In Situ* Time-Resolved
X-Ray Diffraction of Tobermorite Formation Process Under Autoclave
Condition. J. Am. Ceram. Soc..

[85] Matsui K., Kikuma J., Tsunashima M., Ishikawa T., Matsuno S., Ogawa A., Sato M. (2011). In Situ Time-Resolved X-Ray Diffraction
of Tobermorite Formation in Autoclaved Aerated Concrete: Influence
of Silica Source Reactivity and Al Addition. Cem. Concr. Res..

[86] Mitsuda T., Taylor H. F. W. (1975). Influence of
Aluminium on the Conversion of Calcium
Silicate Hydrate Gels into 11 Å Tobermorite at 90°C and
120°C. Cem. Concr. Res..

[87] Shaw S., Clark S. M., Henderson C. M. B. (2000). Hydrothermal
Formation of the Calcium
Silicate Hydrates, Tobermorite (Ca_5_Si_6_O_16_(OH)_2_·4H_2_O) and Xonotlite (Ca_6_Si_6_O_17_(OH)_2_): An in Situ
Synchrotron Study. Chem. Geol..

[88] Vyazovkin S., Burnham A. K., Criado J. M., Pérez-Maqueda L. A., Popescu C., Sbirrazzuoli N. (2011). ICTAC Kinetics Committee Recommendations
for Performing Kinetic Computations on Thermal Analysis Data. Thermochim. Acta.

[89] Galwey, A. K. Thermal Decomposition of Ionic Solids. In Studies in physical and theoretical chemistry, 1st ed.; Elsevier: Amsterdam, 1999.

[90] Cruciani G., Gualtieri A. (1999). Dehydration
Dynamics of Analcime by in Situ Synchrotron
Powder Diffraction. Am. Mineral..

[91] Gualtieri A. F. (2000). Accuracy
of XRPD QPA Using the Combined Rietveld–RIR Method. J. Appl. Crystallogr..

[92] Colville A. A., Geller S. (1971). The Crystal Structure
of Brownmillerite, Ca_2_FeAlO_5_. Acta Crystallogr., Sect.
B.

[93] Ondrus P., Veselovsky F., Gabasova A., Hlousek J., Srein V., Vavrin I., Skala R., Sejkora J., Drabek M. (2003). Primary Minerals
of the Jachymov Ore District. J. Geosci..

[94] Alberti A., Galli E., Vezzalini G., Passaglia E., Zanazzi P. F. (1982). Position of Cations and Water Molecules in Hydrated
Chabazite. Natural and Na-, Ca-, Sr- and K-Exchanged Chabazites. Zeolites.

[95] Yamnova N. A., Zubkova N. V., Eremin N. N., Zadov A. E., Gazeev V. M. (2011). Crystal
Structure of Larnite β-Ca2SiO4 and Specific Features of Polymorphic
Transitions in Dicalcium Orthosilicate. Crystallogr.
Rep..

[96] Blasi A., Brajkovic A., De Pol Blasi C., Foord E. E., Martin R. F., Zanazzi P. F. (1984). Structure refinement
and genetic aspects of a microcline
overgrowth on amazonite from Pikes Peak batholith, Colorado, U.S.A. Bull. Minéralogie.

[97] Brigatti M. F., Guidotti C. V., Malferrari D., Sassi F. P. (2008). Single-Crystal X-Ray
Studies of Trioctahedral Micas Coexisting with Dioctahedral Micas
in Metamorphic Sequences from Western Maine. Am. Mineral..

[98] Hazen R. M. (1976). Effects
of Temperature and Pressure on the Cell Dimension and X-Ray Temperature
Factors of Periclase. Am. Mineral..

[99] Gatta G. D., Cappelletti P., Rotiroti N., Slebodnick C., Rinaldi R. (2009). New Insights into the
Crystal Structure and Crystal
Chemistry of the Zeolite Phillipsite. Am. Mineral..

[100] Harlow G. E. (1982). The Anorthoclase
Structures: The Effects of Temperature
and Composition. Am. Mineral.

[101] Le Page Y., Donnay G. (1976). Refinement of the Crystal
Structure
of Low-Quartz. Acta Crystallogr., Sect. B.

[102] Yamnova N. A., Khomyakov A. P., Zlykhenskaya I. V. (2000). Refinement
of the Crystal Structure of Sanidine-like Feldspar. Crystallogr. Rep..

[103] Chakoumakos B. C., Pracheil B. M., Koenigs R. P., Bruch R. M., Feygenson M. (2016). Empirically Testing Vaterite Structural
Models Using
Neutron Diffraction and Thermal Analysis. Sci.
Rep..

[104] Passaglia E., Rinaldi R. (1984). Katoite, a new member
of the Ca_3_Al_2_(SiO_4_)_3_-Ca_3_Al_2_(OH)_12_ series and a new nomenclature
for
the hydrogrossular group of minerals. Bull.
Minéralogie.

[105] Rios C., Williams C., Fullen M. (2009). Hydrothermal Synthesis
of Hydrogarnet and Tobermorite at 175 °C from Kaolinite and Metakaolinite
in the CaO–Al_2_O_3_–SiO_2_–H_2_O System: A Comparative Study. Appl. Clay Sci..

[106] Klimesch D. S., Ray A. (1999). DTA-TG Study of the
CaO-SiO_2_-H2O and CaO-Al_2_O_3_-SiO_2_-H_2_O Systems Under Hydrothermal Conditions. J.
Therm. Anal. Calorim..

[107] Myers R. J., Bernal S. A., San Nicolas R., Provis J. L. (2013). Generalized Structural Description of Calcium–Sodium
Aluminosilicate Hydrate Gels: The Cross-Linked Substituted Tobermorite
Model. Langmuir.

[108] Kikuma J., Tsunashima M., Ishikawa T., Matsuno S., Ogawa A., Matsui K., Sato M. (2009). Hydrothermal Formation
of Tobermorite Studied by *in Situ* X-Ray Diffraction
under Autoclave Condition. J. Synchrotron Radiat..

[109] Bernstein S. (2011). Determination
of Reaction Kinetics and Mechanisms of
1.13 NM Tobermorite by in-Situ Neutron Diffraction. Ludwig-Maximilians-Universität München.

[110] Wu Y., Pan X., Li Q., Yu H. (2020). Crystallization and
Phase Transition of Tobermorite Synthesized by Hydrothermal Reaction
from Dicalcium Silicate. Int. J. Appl. Ceram.
Technol..

[111] Földvári, M. Handbook Of Thermogravimetric System Of Minerals And Its Use In Geological Practice; Geological Inst. of Hungary: Budapest, 2011.

[112] Kissinger H. E. (1957). Reaction Kinetics in Differential Thermal Analysis. Anal. Chem..

[113] Malferrari, D. ; Di Giuseppe, D. ; Scognamiglio, V. ; Gualtieri, A. Commercial Brucite, a Worldwide Used Raw Material Deemed Safe, Can Be Contaminated by Asbestos. Period. Mineral., 2021, 90 3. 10.13133/2239-1002/17384.

[114] Komarneni S., Roy R., Roy D. M., Fyfe C. A., Kennedy G. J. (1985). Al-Substituted Tobermorite—The Coordination
of Aluminum as Revealed by Solid-State 27Al Magic Angle Spinning (MAS)
NMR. Cem. Concr. Res..

[115] Mostafa N. Y., Shaltout A. A., Omar H., Abo-El-Enein S. A. (2009). Hydrothermal
Synthesis and Characterization of Aluminium and Sulfate Substituted
1.1nm Tobermorites. J. Alloys Compd..

[116] Black L., Stumm A., Garbev K., Stemmermann P., Hallam K. R., Allen G. C. (2005). X-Ray Photoelectron Spectroscopy
of Aluminium-Substituted Tobermorite. Cem. Concr.
Res..

[117] Li X., Zhang H., Zhan H., Tang Y. (2023). Structural and Mechanical
Properties of Doped Tobermorite. Nanomaterials.

