# Supplementary material for: Kinetic Investigation of Tobermorite Synthesis for the Recovery of Carcinogenic Respirable Crystalline Silica (RCS)
Source: ACS Omega. 2025 Oct 24;10(43):51284–96. doi: 10.1021/acsomega.5c06547 (PMC12593074; doi:10.1021/acsomega.5c06547)
Supplement: Supplementary file 1 [file ao5c06547_si_001.pdf]

SUPPORTING INFORMATION
SUPPLEMENTARY DATA AND MATERIAL FOR

Kinetic investigation of tobermorite synthesis for the recovery of carcinogenic respirable crystalline silica (RCS)

Daniele Malferrari[a,b,*], *Giulio Galamini,*[a*], *Maddalena Bernini*[a], *Riccardo Fantini*[a], *Giulia Malvolti*[a], *Alessandro F. Gualtieri*[a,b]

[a] Department of Chemical and Geological Sciences, University of Modena and Reggio Emilia, Via G. Campi 103, I-41125, Modena, Italy

[b] Inter-Departmental Research and Innovation Centre on Construction and Environmental Services of the University of Modena and Reggio Emilia, Via P. Vivarelli 10, I-41125 Modena, Italy

[*] daniele.malferrari@unimore.it

[*] giulio.galamini@unimore.it

Analytical methods and experimental conditions

Particle size analysis was carried out to characterize QD using a Malvern 3000 equipped with a Hydro EV dispersion unit according to the reference standard ISO 13320 (ISO 13320: 2009 "Particle size analysis - Laser diffraction methods "). The measurable range through the instrument is between 0.01 and 3500 μm. The optical model used is the Mie model, under the following optical properties: Zeolite A Refractive Index 1.446; Zeolite Absorption Index 0.010; Dispersant Name Water; Dispersant Refractive Index 1.330. The analyses were performed on three different aliquots for each sample, dispersed by stirring at 2500 rpm. For each sub-sample five replicas were performed, in accordance with ISO 13320 and within ISO Limits.

X-ray fluorescence (XRF) was used to obtain chemical analyses of each solid sample and were performed using the Panalytical Zetium sequential wavelength dispersive spectrometer (WDS), operating under vacuum conditions, which allows determination of concentrations of elements from fluorine (F) to uranium (U), equipped with 3 kW Rh tube and 5 analyzer crystals (LiF220, LiF200, Ge, PE, PX1) operated alternately depending on the element to be analyzed. Measurements are made on tablets obtained by pressing at 15 t for 3 min a definite amount of finely ground powder on a carrier formed from boric acid.

Mineralogical analyses were achieved through X-ray powder diffraction measurements (XRPD) taken at room temperature using a Philips X'Pert PRO diffractometer equipped with a first-generation Real Time Multiple Strip detector. Experimental conditions were: incident beam, Cu Kα radiation at 40 kV and 30 mA; Soller slits, 0.02 rad; anti-scatter mask, 20 mm; anti-scatter slit, 1/4°; divergence slit, 1/4°. Diffracted beam: anti-scatter mask, 5.0 mm; filter, nickel; Soller slits, 0.02 rad; integration time, 240 s in continuous scan (length of 2.12 °2θ corresponding to a step size of 0.0170 °2θ per s). Measurements were collected in the range 3-90 °2θ. NIST SRM 676a (alumina powder, corundum structure) was used as the internal standard (10 % in weight). After mild drying to remove humidity water, sample and standard were efficiently ground and homogenized before measurement. Samples were side-loaded to avoid preferred orientations (tobermorite and illite have a lamellar morphology), but these effects were also minimized during the refinement. The quantitative phase analyses (QPA) was performed by the Rietveld method using the General Structure Analysis System (GSAS) software package and EXPGUI as the graphical user interface and following the protocol reported in Gualtieri et al. (2019). [1–3] The structural models used for the full-profile fit refinement are given in Table S1 where error on each measurement is also indicated. Background was fitted with

Function 1 in GSAS and a March-Dollase function has been applied to minimize preferred orientation effect. Quantitative refinement of corundum was used to calculate the amount of amorphous.

Scanning Electron Microscopy with Energy Dispersive X-ray analyses (SEM-EDX) measurements were carried out on about 0.5 mg of powder previously suspended in 1 mL of Millipore water and ultrasonicated for 10 min to prompt the separation of the particles. After the suspension was dispersed on an aluminium stub previously covered with carbon-conductive adhesive tape, dried at room temperature and then carbon metallized. Carbon-coated materials was observed with the Scanning Electron Microscope (SEM) JEOL JSM–6010PLUS/LA InTouchScope, equipped with an Energy Dispersive X–ray (EDX) detector. SEM-EDX measurements were performed in high vacuum with an accelerating voltage between 5 and 20 keV both for imaging and elemental analyses.

Thermogravimetric (TGA) and differential thermal analyses (DTA) were performed using a Seiko SSC 5200 thermal analyzer paired with a quadrupole **mass spectrometer** (ESS, GeneSys Quadstar 422) to monitor the **gases released during thermal reactions** (MSEGA). Gas sampling by the spectrometer was achieved through an inert, fused silica capillary, which was heated to prevent gas condensation. The experimental conditions were as follows: heating rate of 20 °C/min, temperature range from 25 to 1000 °C, data collection every 0.5 seconds, and purging gas of ultrapure helium with a flow rate of 100 µL/min. Mass spectrometry was conducted in multiple ion detection mode, measuring the m/z ratios of 18 and 44 to detect H_2O and CO_2 emissions, respectively. A secondary electron multiplier detector, set to 900 V, was used with a 1-second integration time for each mass measurement.

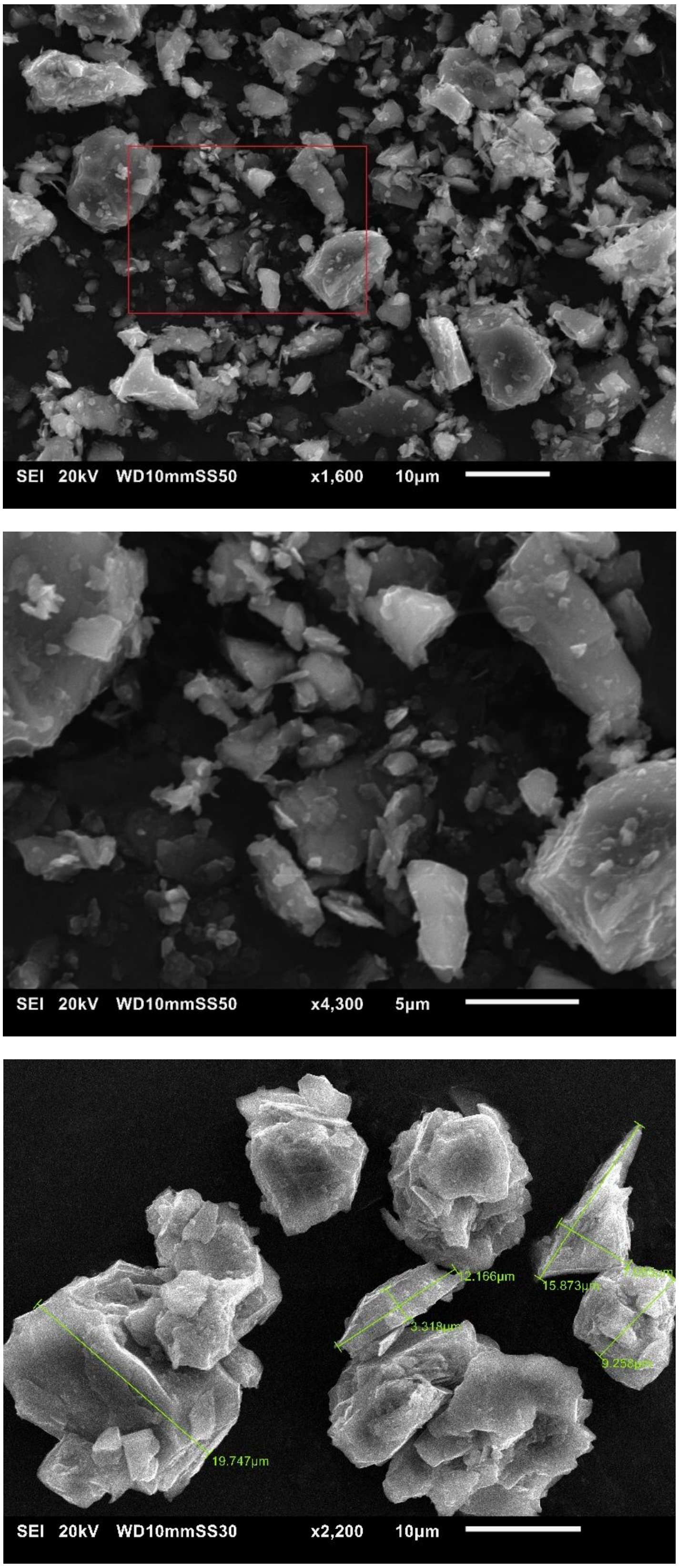

Figure S1. Selected SEM images of the QD sample (top) with a magnification of the area in the red box (centre) and measurements of some grains (bottom) showing the occurrence of quartz particles which are respirable and could reach the alveolar regions of the lungs. Small lamellar crystals on the grain surface are muscovite (see Table 1 in the main text).

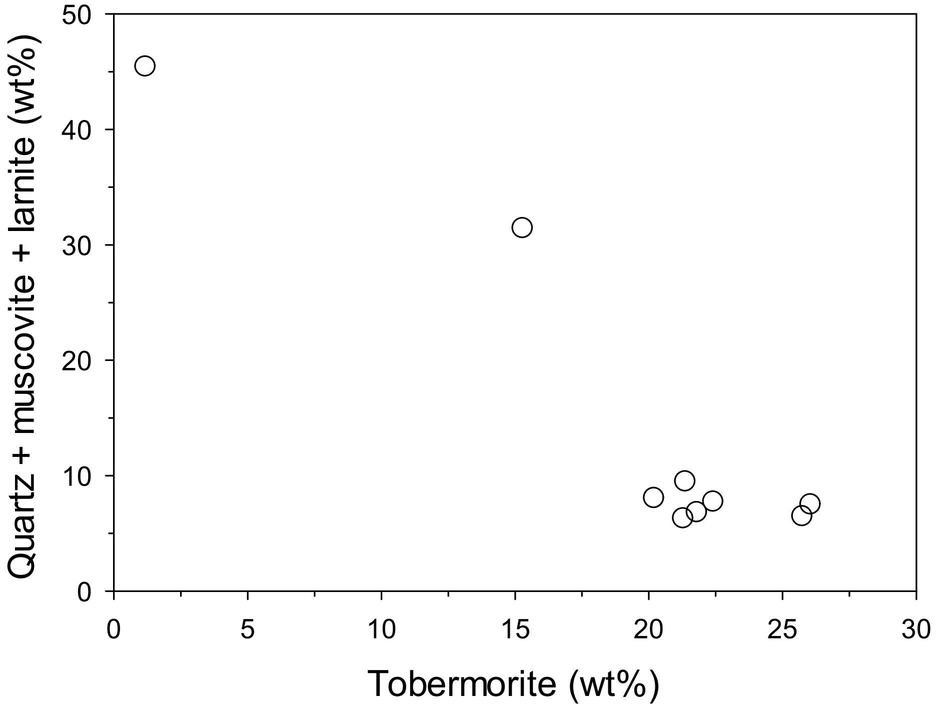

Figure S2. Inverse correlation between the sum of the amounts of larnite, muscovite and quartz compared to that of tobermorite from M1 at 140 °C.

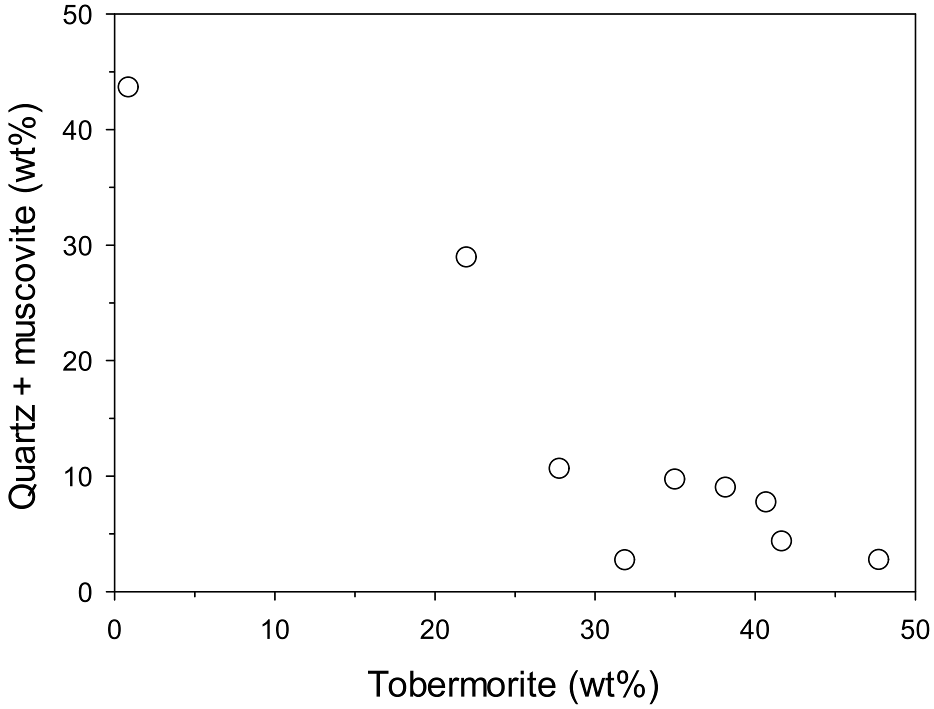

Figure S3. Inverse correlation between the amount of quartz compared to that of tobermorite from M2 at 140 °C.

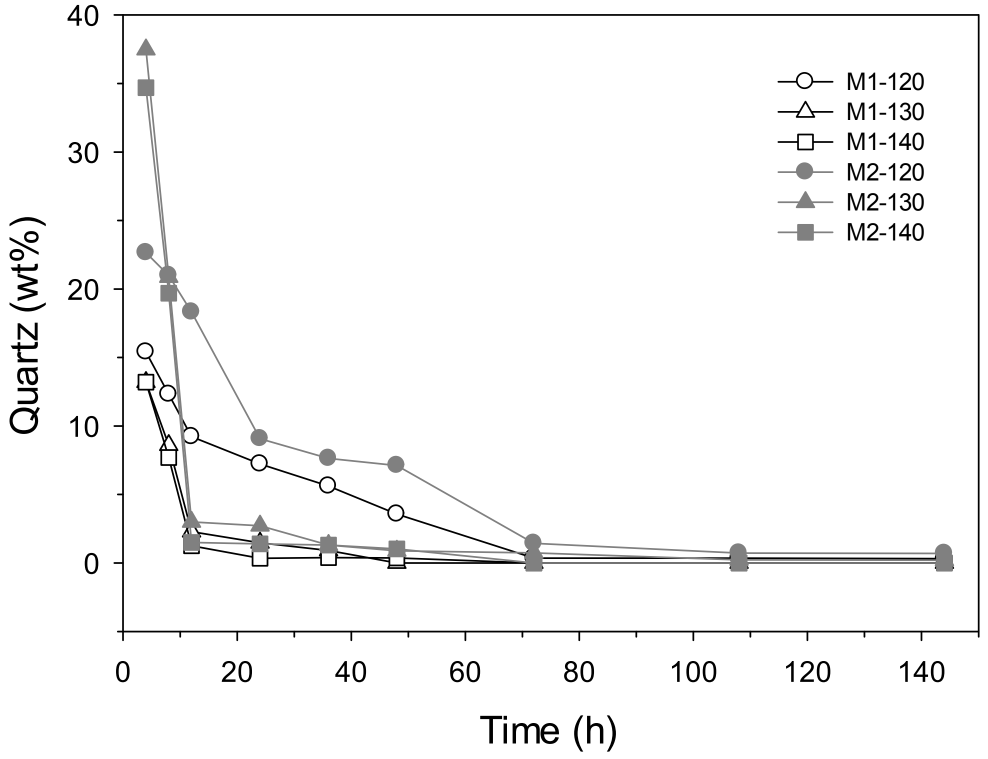

Figure S4. Quartz consumption for M1 and M2 as temperature and time change.

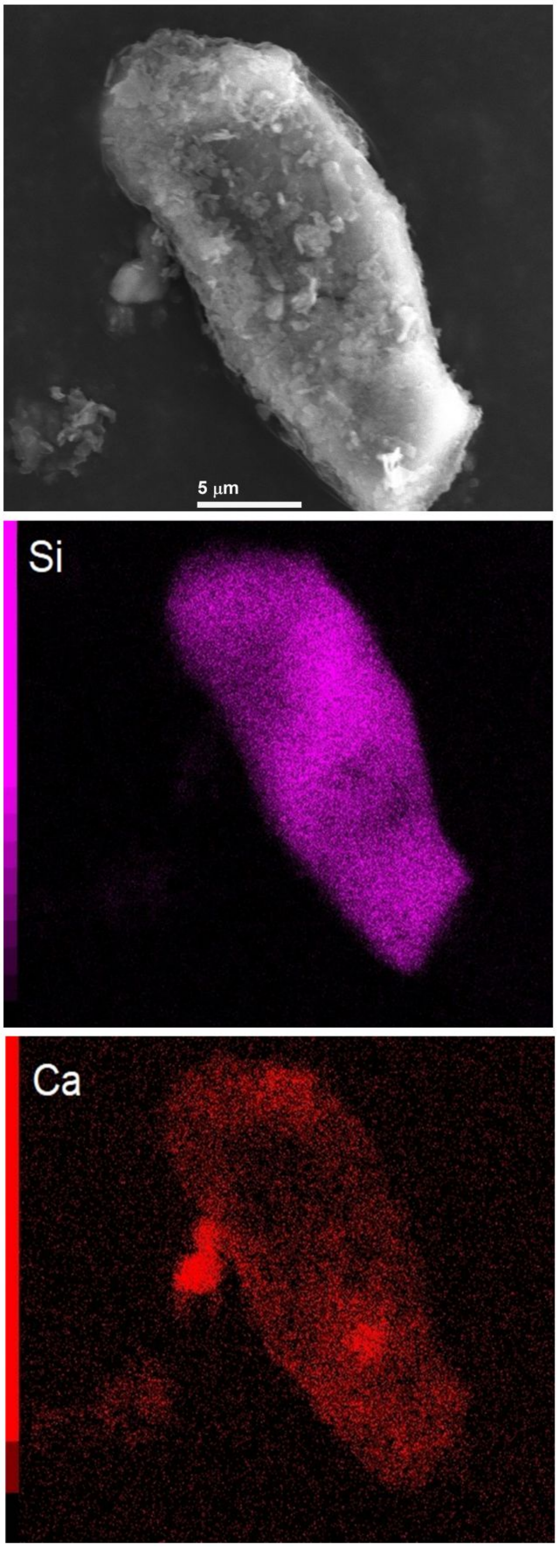

Figure S5. SEM image and EDX analysis (chemical maps for Si and Ca distribution) of a quartz grain during dissolution and transformation into tobermorite in M2-12H-140 sample. The lamellar structures at the edges, where the calcium is most concentrated, are crystals of growing tobermorite.

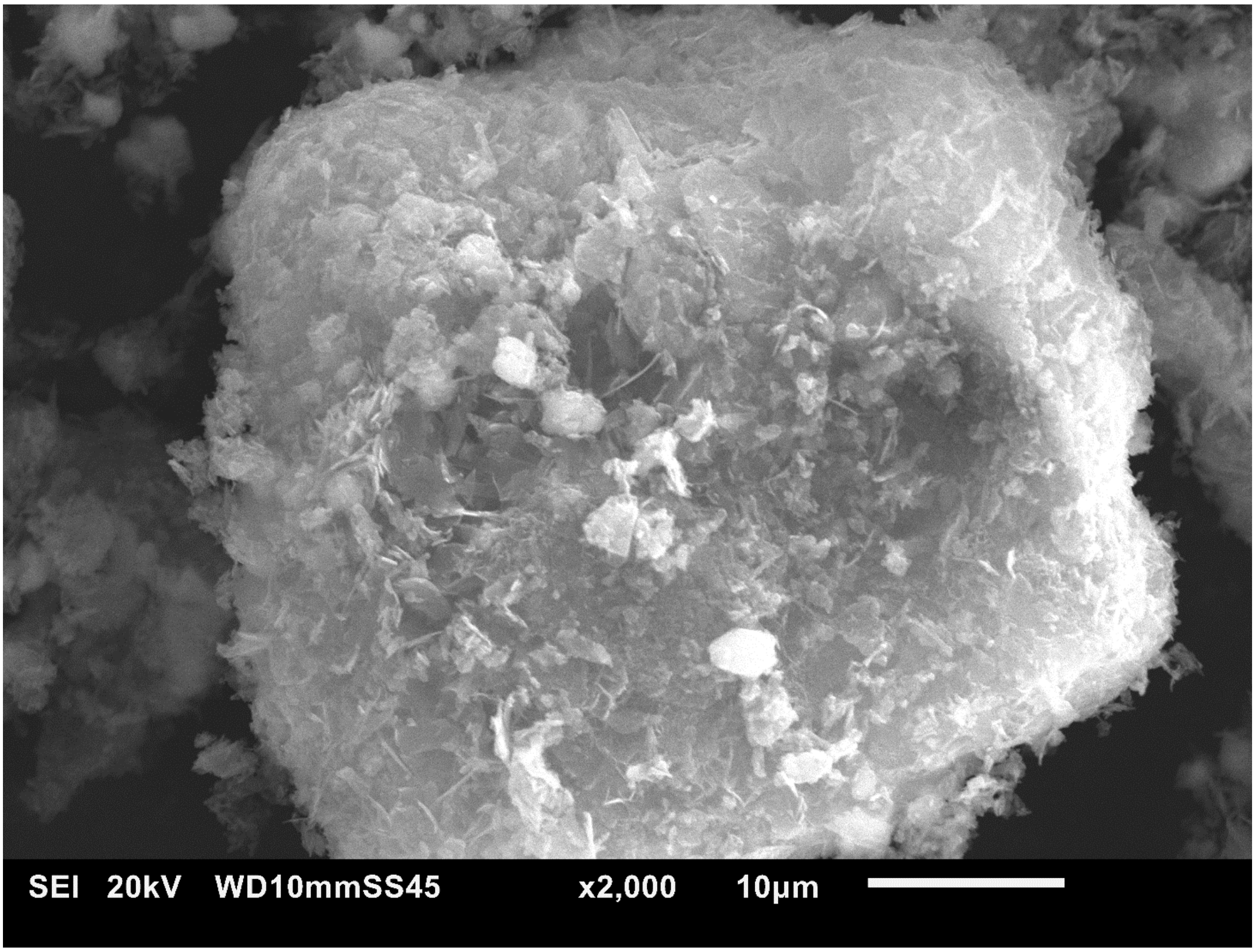

Figure S6. SEM image of lamellar crystals of tobermorite in near-complete pseudomorphosis on a quartz grain in sample M1-48H-140.

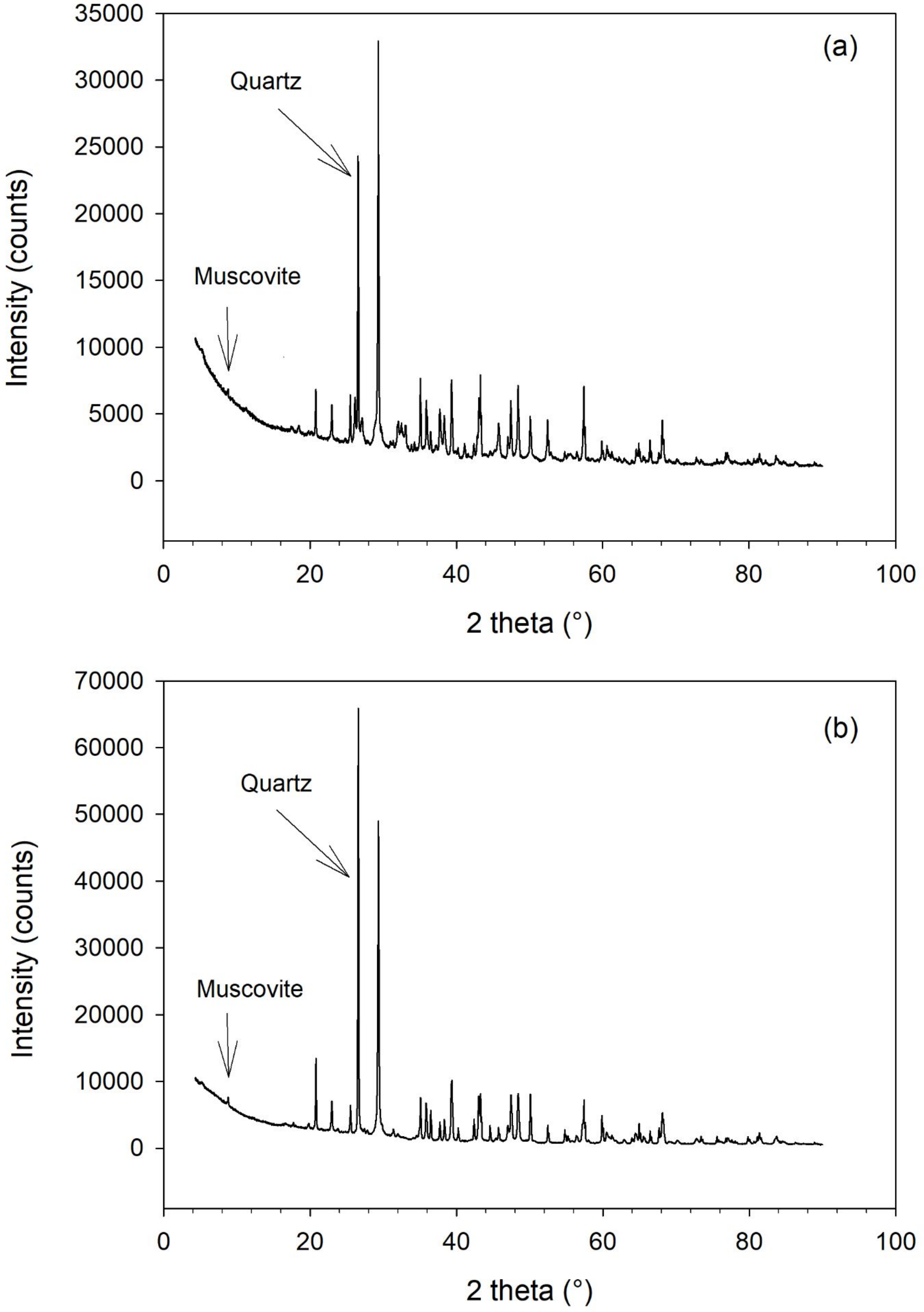

Figure S7. XRPD pattern from M1 (a) and M2 (b) after 144 h at 110 °C. It should be noted the absence of signals related to tobermorite and the presence of strong signal from quartz and, at low angle, the signal from muscovite, both occurring in the source materials.

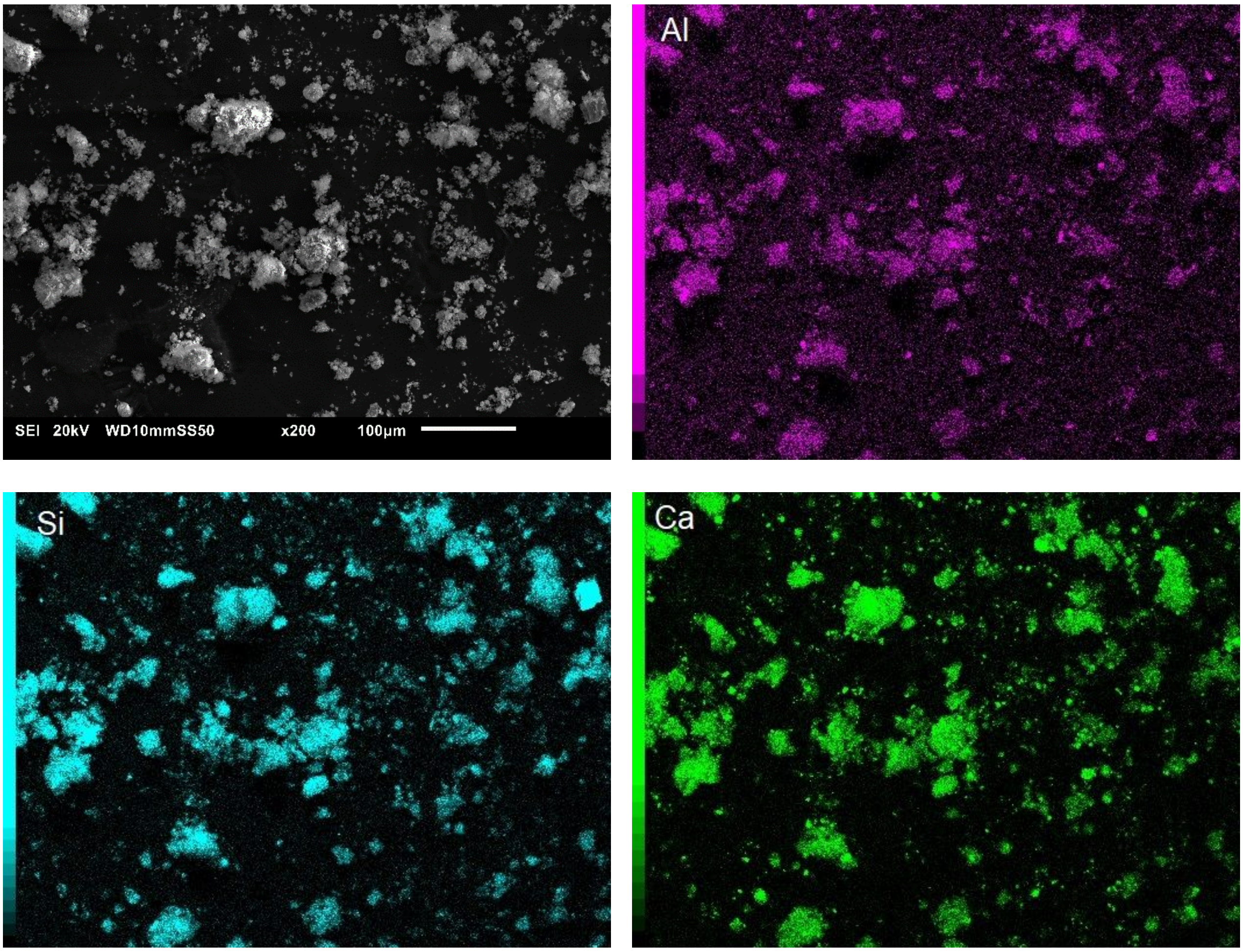

Figure S8. SEM image and EDX chemical maps of sample M1-48H-140. For the dimension scale see the bar in the SEI image.

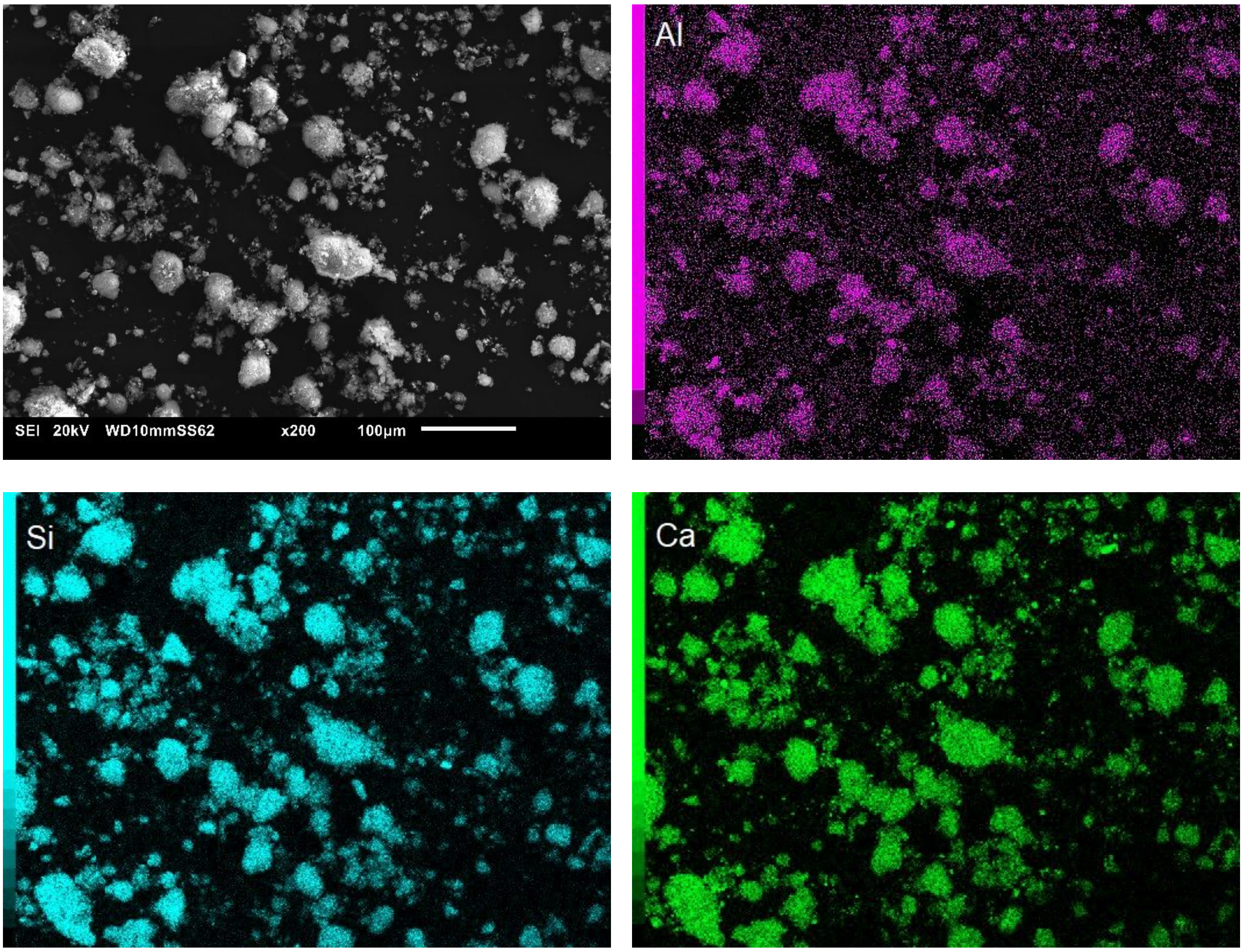

Figure S9. SEM image and EDX chemical maps of sample M2-144H-140. For the dimension scale see the bar in the SEI image.

Table S1. Mineralogical (weight %) composition of M1(S1a) and M2 (S1b) products. The standard deviation σ_Q (values in parenthesis) of the weight percentage Q of each phase was calculated using the values obtained in the output file after the quantitative refinement by GSAS software, and the formula $\sigma_Q = \{[(\sigma_a/a)^2 + (\sigma_b/b)^2]^{1/2}\}$ Q, where *a* and *b* are the two variables most affecting Q values and refer, respectively, to the weight fraction of the phase and the internal standard, whereas σ_a and σ_b are their standard deviations.[4] The literature reference for the structural models used in QPA are: aragonite,[5] brucite,[6] calcite,[7] katoite,[8] larnite,[9] muscovite,[10] portlandite,[11] quartz,[12] tobermorite,[13] vaterite.[14] The symbol "-" means absent or non-quantifiable by QPA.

Table S1a

	**Time (h)**	**Aragonite**	**Brucite**	**Calcite**	**Katoite**	**Larnite**	**Muscovite**	**Portland.**	**Quartz**	**Toberm.**	**Vaterite**	**Amorph.**	χ^2	R_p **(%)**	R_{wp} **(%)**
**M1-120**	**4**	8.8(3)	-	9.3(2)	-	27.6(1)	3.9(2)	-	15.4(7)	-	6.6(6)	28.4(8)	*5.336*	*0.0358*	*0.0432*
	**8**	9.6(2)	-	12.9(3)	2.8(2)	16.8(2)	3.8(4)	-	12.3(6)	-	8.4(5)	33.4(7)	*6.932*	*0.0373*	*0.0501*
	**12**	10.2(3)	-	17.9(5)	3.6(3)	9.7(3)	3.9(6)	-	9.2(3)	7.2(4)	13.6(6)	24.7(9)	*5.664*	*0.0341*	*0.0479*
	**24**	10.9(1)	-	20.4(2)	4.5(2)	7.3(1)	3.8(3)	-	7.2(7)	7.5(2)	8.4(3)	30.0(6)	*5.410*	*0.0343*	*0.0477*
	**36**	11.2(4)	-	19.2(2)	4.9(3)	6.1(7)	2.9(3)	-	5.6(4)	9.7(3)	13.2(3)	27.2(5)	*4.525*	*0.0423*	*0.0317*
	**48**	14.4(4)	-	18.5(2)	4.3(3)	4.1(7)	1.8(3)	-	3.6(4)	10.5(3)	9.3(4)	33.5(5)	*6.104*	*0.0365*	*0.0494*
	**72**	1.9(5)	-	21.3(2)	5.7(2)	2.9(9)	1.6(2)	-	0.4(3)	16.7(2)	15.8(4)	33.7(5)	*12.31*	*0.0504*	*0.0708*
	**108**	4.0(1)	-	23.2(2)	3.4(1)	3.1(1)	1.5(2)	-	0.4(3)	18.4(2)	5.2(2)	40.8(4)	*8.223*	*0.0433*	*0.0581*
	**144**	11.2(1)	-	18.2(1)	5.1(1)	3.3(1)	0.9(5)	-	0.3(4)	21.9(2)	8.0(3)	31.1(4)	*6.535*	*0.0371*	*0.0500*
**M1-130**	**4**	1.6(5)	-	11.3(1)	-	22.6(2)	3.9(2)	-	13.2(5)	-	-	47.4(7)	*9.814*	*0.0437*	*0.0625*
	**8**	1.9(6)	1.2(3)	15.6(1)	4.4(3)	15.4(2)	3.1(2)	-	8.6(4)	-	-	49.8(8)	*9.472*	*0.0428*	*0.0603*
	**12**	2.1(7)	2.0(4)	20.3(2)	7.1(3)	7.3(1)	2.0(2)	-	2.3(4)	16.2(2)	-	40.7(7)	*9.746*	*0.0499*	*0.0598*
	**24**	1.4(6)	1.3(4)	22.2(3)	6.9(2)	5.5(2)	1.6(4)	-	1.5(5)	17.6(1)	-	42.0(7)	*9.129*	*0.0417*	*0.0581*
	**36**	4.2(4)	2.2(3)	24.2(3)	6.7(3)	5.2(3)	1.1(3)	-	0.9(3)	18.9(2)	-	36.6(8)	*9.991*	*0.0512*	*0.0616*
	**48**	4.0(5)	2.0(4)	21.0(2)	4.3(3)	5.0(2)	-	-	-	18.8(3)	-	44.9(6)	*10.28*	*0.0468*	*0.0320*
	**72**	4.1(6)	2.4(4)	19.2(1)	3.0(2)	4.2(1)	-	-	-	19.3(2)	-	47.8(8)	*9.384*	*0.0423*	*0.0629*
	**108**	4.1(3)	1.8(3)	21.4(2)	2.6(3)	4.4(2)	-	-	-	18.5(2)	-	47.2(6)	*10.93*	*0.0476*	*0.0664*
	**144**	5.9(1)	-	19.3(1)	1.6(2)	3.6(2)	-	-	-	18.2(2)	-	51.4(8)	*12.25*	*0.0498*	*0.0704*
**M1-140**	**4**	1.3(1)	1.3(4)	11.5(1)	7.5(2)	29.4(2)	2.8(3)	-	13.2(1)	1.2(3)	-	31.8(4)	*2.417*	*0.0678*	*0.0914*
	**8**	1.9(2)	2.7(5)	11.9(1)	8.6(2)	21.1(3)	2.6(3)	-	7.7(1)	15.3(2)	-	28.2(3)	*2.004*	*0.0630*	*0.0821*
	**12**	3.3(1)	2.1(8)	21.0(2)	8.9(7)	6.6(1)	1.6(2)	-	1.3(1)	21.4(2)	-	33.8(4)	*7.171*	*0.0380*	*0.0524*
	**24**	4.2(2)	2.3(5)	23.7(2)	6.4(5)	6.4(3)	1.3(3)	-	0.3(3)	20.2(3)	-	35.2(4)	*7.048*	*0.0392*	*0.0528*
	**36**	4.2(2)	2.2(4)	24.2(3)	6.7(5)	6.2(2)	1.1(3)	-	0.4(2)	22.4(3)	-	32.6(4)	*5.761*	*0.0358*	*0.0475*
	**48**	4.7(2)	2.2(4)	24.8(3)	6.7(4)	6.2(2)	0.9(4)	-	0.4(2)	26.1(3)	-	28.0(6)	*6.646*	*0.0516*	*0.0385*
	**72**	1.7(3)	2.6(5)	24.5(2)	6.1(5)	6.4(2)	-	-	-	25.8(2)	-	32.9(6)	*6.654*	*0.0349*	*0.0487*
	**108**	1.1(1)	2.0(4)	20.0(3)	2.0(4)	6.3(3)	-	-	-	21.3(2)	-	47.3(5)	*10.26*	*0.0464*	*0.0643*
	**144**	4.0(1)	1.2(3)	21.2(1)	2.2(2)	6.8(1)	-	-	-	21.8(2)	-	42.8(6)	*8.125*	*0.0418*	*0.0569*

Table S1b

	Time (h)	Aragonite	Brucite	Calcite	Katoite	Larnite	Muscovite	Portland.	Quartz	Toberm.	Vaterite	Amorph.	χ^2	R_p (%)	R_{wp} (%)
**M2-120**	**4**	-	-	8.6(1)	-	-	10.3(1)	37.7(2)	22.7(6)	-	-	20.7(3)	*8.412*	*0.0416*	*0.0616*
	**8**	-	-	7.4(1)	-	-	10.6(1)	29.0(2)	21.0(6)	-	-	32.0(4)	*8.514*	*0.0433*	*0.0608*
	**12**	1.0(3)	-	34.3(3)	-	-	6.7(2)	-	18.3(5)	9.7(2)	2.5(4)	27.5(3)	*8.755*	*0.0463*	*0.0646*
	**24**	1.8(3)	-	38.7(2)	-	-	7.4(2)	-	9.1(4)	11.9(2)	3.8(4)	27.3(3)	*5.876*	*0.0353*	*0.0501*
	**36**	7.4(5)	-	28.9(2)	-	-	7.1(2)	-	7.6(6)	26.4(2)	7.9(5)	14.7(4)	*8.258*	*0.0430*	*0.0595*
	**48**	8.3(2)	-	26.8(2)	-	-	6.7(2)	-	7.1(9)	19.0(2)	9.1(7)	23.0(4)	*8.199*	*0.0439*	*0.0623*
	**72**	4.9(5)	-	24.9(2)	-	-	7.6(1)	-	1.4(3)	25.1(3)	5.9(5)	30.2(4)	*6.334*	*0.0367*	*0.0510*
	**108**	2.1(4)	-	19.8(2)	-	-	7.0(2)	-	0.7(3)	33.3(3)	6.6(3)	30.5(8)	*10.43*	*0.0500*	*0.0697*
	**144**	5.2(9)	-	16.7(2)	-	-	6.3(2)	-	0.7(4)	38.5(3)	6.3(2)	26.3(4)	*9.329*	*0.0439*	*0.0630*
**M2-130**	**4**	-	-	8.0(1)	-	-	9.6(1)	35.1(2)	37.5(6)	-	-	9.8(5)	*11.79*	*0.0486*	*0.0723*
	**8**	-	-	6.8(1)	-	-	9.8(2)	18.4(2)	20.9(6)	-	-	44.1(5)	*12.01*	*0.0501*	*0.0616*
	**12**	0.9(3)	-	21.8(2)	-	-	9.1(1)	-	3.0(4)	20.8(2)	5.4(3)	39.0(7)	*11.28*	*0.0493*	*0.0707*
	**24**	2.3(3)	-	23.2(2)	-	-	6.9(2)	-	2.7(4)	22.9(2)	7.1(3)	34.9(5)	*12.40*	*0.0545*	*0.0783*
	**36**	1.4(3)	-	23.1(1)	-	-	7.6(1)	-	1.3(3)	35.2(2)	7.7(4)	23.7(6)	*11.61*	*0.0502*	*0.0720*
	**48**	2.4(1)	-	23.4(1)	-	-	6.6(2)	-	0.9(3)	27.7(1)	8.2(3)	30.8(5)	*8.428*	*0.0434*	*0.0615*
	**72**	2.7(2)	-	14.7(1)	-	-	4.1(2)	-	0.7(3)	27.8(2)	7.9(3)	42.1(5)	*10.01*	*0.0468*	*0.0667*
	**108**	2.5(3)	-	17.8(2)	-	-	4.1(2)	-	0.2(3)	37.9(3)	7.4(3)	30.1(7)	*13.76*	*0.0549*	*0.0781*
	**144**	2.5(3)	-	17.6(2)	-	-	3.5(2)	-	0.2(1)	42.6(3)	6.9(3)	26.7(7)	*13.44*	*0.0516*	*0.0755*
**M2-140**	**4**	-	-	7.4(1)	-	-	8.9(2)	32.5(3)	34.7(9)	0.9(4)	-	15.6(9)	*5.126*	*0.0532*	*0.0725*
	**8**	-	-	6.4(1)	-	-	9.2(4)	17.4(2)	19.7(9)	22.0(2)	-	25.3(9)	*4.113*	*0.0488*	*0.0672*
	**12**	0.9(2)	-	21.8(2)	-	-	9.1(2)	-	1.5(2)	27.8(2)	5.4(1)	33.5(4)	*5.875*	*0.0493*	*0.0582*
	**24**	1.7(3)	-	22.3(1)	-	-	8.3(2)	-	1.4(2)	35.0(3)	8.1(2)	23.2(4)	*5.521*	*0.0401*	*0.0605*
	**36**	1.4(3)	-	23.1(1)	-	-	7.6(2)	-	1.3(4)	38.2(3)	-	28.4(5)	*6.161*	*0.0670*	*0.0719*
	**48**	1.5(1)	-	20.7(2)	-	-	6.7(2)	-	1.0(7)	40.7(2)	-	29.4(4)	*6.764*	*0.0417*	*0.0636*
	**72**	2.2(2)	-	18.9(1)	-	-	4.3(2)	-	-	41.7(3)	-	32.9(7)	*5.938*	*0.0483*	*0.0677*
	**108**	1.3(1)	-	17.5(2)	-	-	2.7(3)	-	-	31.9(4)	-	46.6(6)	*6.742*	*0.0516*	*0.0692*
	**144**	2.8(3)	-	18.4(1)	-	-	2.7(3)	-	-	47.8(4)	-	28.3(6)	*5.202*	*0.0510*	*0.0652*

References

(1) Gualtieri, A. F.; Gatta, G. D.; Arletti, R.; Artioli, G.; Ballirano, P.; Cruciani, G.; Guagliardi, A.; Malferrari, D.; Masciocchi, N.; Scardi, P. Quantitative Phase Analysis Using the Rietveld Method: Towards a Procedure for Checking the Reliability and Quality of the Results. *Period. Mineral.* **2019**, *88* (2), 147–151. https://doi.org/10.2451/2019PM870.

(2) Toby, B. H. EXPGUI, a Graphical User Interface for GSAS. *J. Appl. Crystallogr.* **2001**, *34* (2), 210–213. https://doi.org/10.1107/S0021889801002242.

(3) Larson, A. C.; Von Dreele, R. B. *General Structure Analysis System (GSAS)*; Los Alamos National Laboratory, Report LAUR 86-748, 1994.

(4) Young, H. D. *Statistical Treatment of Experimental Data*, McGraw-Hill Book Company, New York.; 1962.

(5) Villiers, J. P. R. D. Crystal Structures of Aragonite, Strontianite, and Witherite. *Am. Mineral.* **1971**, *56* (5–6), 758–767.

(6) Catti, M.; Ferraris, G.; Hull, S.; Pavese, A. Static Compression and H Disorder in Brucite, Mg(OH)2, to 11 GPa: A Powder Neutron Diffraction Study. *Phys. Chem. Miner.* **1995**, *22* (3). https://doi.org/10.1007/BF00202300.

(7) Ondrus, P.; Veselovsky, F.; Gabasova, A.; Hlousek, J.; Srein, V.; Vavrin, I.; Skala, R.; Sejkora, J.; Drabek, M. Primary Minerals of the Jachymov Ore District. *J. Geosci.* **2003**, *48* (3–4), 19–147.

(8) Ferro, O.; Galli, E.; Papp, G.; Quartieri, S.; Szakáll, S.; Vezzalini, G. A New Occurrence of Katoite and Re-Examination of the Hydrogrossular Group. *Eur. J. Mineral.* **2003**, *15* (2), 419–426. https://doi.org/10.1127/0935-1221/2003/0015-0419.

(9) Yamnova, N. A.; Zubkova, N. V.; Eremin, N. N.; Zadov, A. E.; Gazeev, V. M. Crystal Structure of Larnite β-Ca2SiO4 and Specific Features of Polymorphic Transitions in Dicalcium Orthosilicate. *Crystallogr. Rep.* **2011**, *56* (2), 210–220. https://doi.org/10.1134/S1063774511020209.

(10) Brigatti, M. F.; Guidotti, C. V.; Malferrari, D.; Sassi, F. P. Single-Crystal X-Ray Studies of Trioctahedral Micas Coexisting with Dioctahedral Micas in Metamorphic Sequences from Western Maine. *Am. Mineral.* **2008**, *93* (2–3), 396–408. https://doi.org/10.2138/am.2008.2523.

(11) Desgranges, L.; Grebille, D.; Calvarin, G.; Chevrier, G.; Floquet, N.; Niepce, J.-C. Hydrogen Thermal Motion in Calcium Hydroxide: $Ca(OH)_2$. *Acta Crystallogr. B* **1993**, *49* (5), 812–817. https://doi.org/10.1107/S0108768193003556.

(12) Le Page, Y.; Donnay, G. Refinement of the Crystal Structure of Low-Quartz. *Acta Crystallogr. B* **1976**, *32* (8), 2456–2459. https://doi.org/10.1107/S0567740876007966.

(13) Yamazaki, S.; Toraya, H. Determination of Positions of Zeolitic Calcium Atoms and Water Molecules in Hydrothermally Formed Aluminum-Substiqtuted Tobermorite-1.1nm Using Synchrotron Radiation Powder Diffraction Data. *J. Am. Ceram. Soc.* **2001**, *84* (11), 2685–2690. https://doi.org/10.1111/j.1151-2916.2001.tb01071.x.

(14) Chakoumakos, B. C.; Pracheil, B. M.; Koenigs, R. P.; Bruch, R. M.; Feygenson, M. Empirically Testing Vaterite Structural Models Using Neutron Diffraction and Thermal Analysis. *Sci. Rep.* **2016**, *6* (1), 36799. https://doi.org/10.1038/srep36799.
